# Supplementary material for: Synthesis and Biological Evaluation of Small Molecules as Potential Anticancer Multitarget Agents
Source: Int J Mol Sci. 2022 Jun 24;23(13):7049. doi: 10.3390/ijms23137049 (PMC9266368; doi:10.3390/ijms23137049)
Supplement: Supplementary file 1 [file ijms-23-07049-s001.zip › ijms-1719158-supplementary.pdf]

Design of small molecules targeting PD-L1 and VEGFR-2

Alberto Pla-López[1], Raquel Castillo[2], Rocío Cejudo-Marín[3], Olaya García-Pedrero[4], Mariam Bakir-Laso[5], Eva Falomir[1]* and Miguel Carda[1]*

[1] Inorganic and Organic Chemistry Department, Univ. Jaume I, E-12071 Castellón, Spain.

[2] Physical and Analytical Chemistry Department. Univ. Jaume I, E-12071 Castellón, Spain.

[3] Predepartmental Medicine Unit. Jaume I, E-12071 Castellón, Spain.

[4] Inst. Univ. Química Organometálica "Enrique Moles", Centro de Innovación en Química Avanzada, Univ. Oviedo, 33006, Oviedo, Spain.

[5] Inst. Univ. Investig. Ciencias Ambientales de Aragón (IUCA), Univ. Zaragoza, 50009 Zaragoza.

Supporting Information

Contents:

S-2: *Synthetic targets*

S-3/S-3: *Synthesis of precursors*

S-4/S-56: *Graphical NMR spectra of synthetic targets*

Synthetic targets

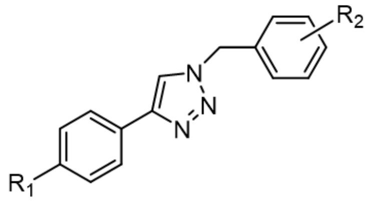

**1**	(52%)	R_1=H,	R_2=H
**2**	(65%)	R_1=H,	R_2=2-Me
**3**	(43%)	R_1=H,	R_2=3-Me
**4**	(55%)	R_1=H,	R_2=4-Me
**5**	(52%)	R_1=H,	R_2=2-MeO
**6**	(63%)	R_1=H,	R_2=3-MeO
**7**	(50%)	R_1=H,	R_2=4-MeO
**8**	(67%)	R_1=H,	R_2=2-Cl
**9**	(59%)	R_1=H,	R_2=3-Cl
**10**	(65%)	R_1=H,	R_2=4-Cl
**11**	(47%)	R_1=H,	R_2=2-Br
**12**	(64%)	R_1=H,	R_2=3-Br
**13**	(65%)	R_1=H,	R_2=4-Br
**14**	(34%)	R_1=NH_2,	R_2=H
**15**	(25%)	R_1=NH_2,	R_2=2-Me
**16**	(42%)	R_1=NH_2,	R_2=3-Me
**17**	(58%)	R_1=NH_2,	R_2=4-Me
**18**	(52%)	R_1=NH_2,	R_2=2-MeO
**19**	(20%)	R_1=NH_2,	R_2=3-MeO
**20**	(38%)	R_1=NH_2,	R_2=4-MeO
**21**	(39%)	R_1=NH_2,	R_2=2-Cl
**22**	(40%)	R_1=NH_2,	R_2=3-Cl
**23**	(71%)	R_1=NH_2,	R_2=4-Cl
**24**	(52%)	R_1=NH_2,	R_2=2-Br
**25**	(73%)	R_1=NH_2,	R_2=3-Br
**26**	(88%)	R_1=NH_2,	R_2=4-Br

Synthesis of precursors

Synthesis of benzyl bromides

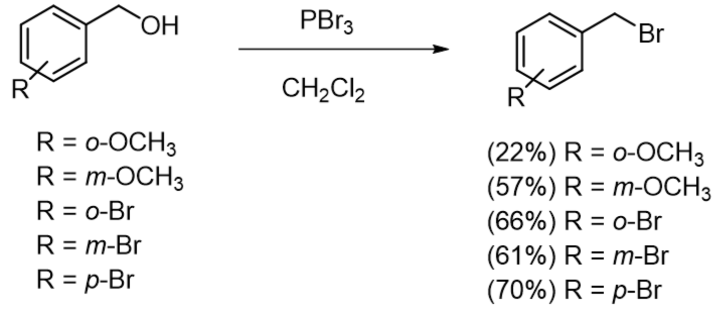

To a solution of the corresponding benzyl alcohol (4 mmol) in CH_2Cl_2 (12 mL) was added PBr_3 dropwise (4 mmol) keeping the reaction flask at 0 ºC by means of an ice bath. Once the addition had finished, the reaction was kept stirring for 2 h at room temperature. Then, the mixture was poured onto a saturated aqueous $NaHCO_3$ solution. Aqueous phase was extracted three times with 10 mL of CH_2Cl_2 and the collected organic phase was washed with brine. Finally, organic phase was dried over anhydrous Na_2SO_4, filtered and concentrated under *vacuum* to afford a residue that was purified on column chromatography using silica gel as stationary phase and a mixture of Hexanes:Ethyl Acetate (95:5) as mobile phase. Benzyl bromides were achieved with yields indicated above.

Synthesis of benzyl azides

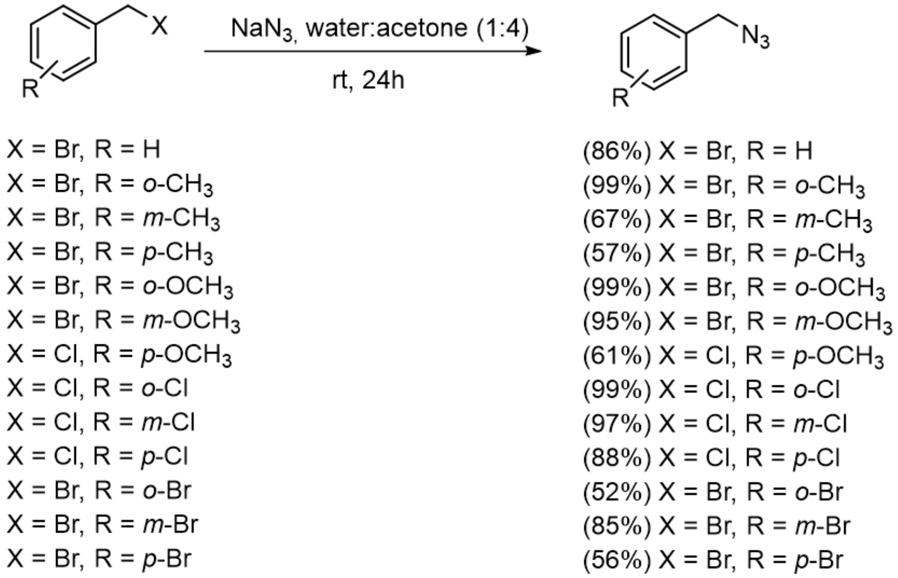

To a solution of NaN_3 (10.5 mmol) in a mixture of water:acetone (1:4, 40 mL) was added the corresponding benzyl bromide or chloride (7 mmol). The reaction mixture was stirred for 24 h at room temperature and then diluted with CH_2Cl_2 (15 mL). Aqueous phase was extracted three times with 10 mL of CH_2Cl_2. Finally, the collected organic phase was dried over anhydrous Na_2SO_4, filtered and evaporated under *vacuum* to afford a residue that was purified on column chromatography using silica gel as stationary phase and a mixture of Hexanes:Ethyl Acetate (95:5) as mobile phase. Benzyl azides were achieved with yields indicated above.

Graphical NMR spectra of synthetic targets

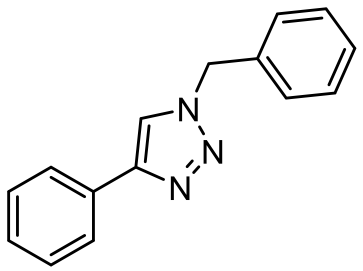

1

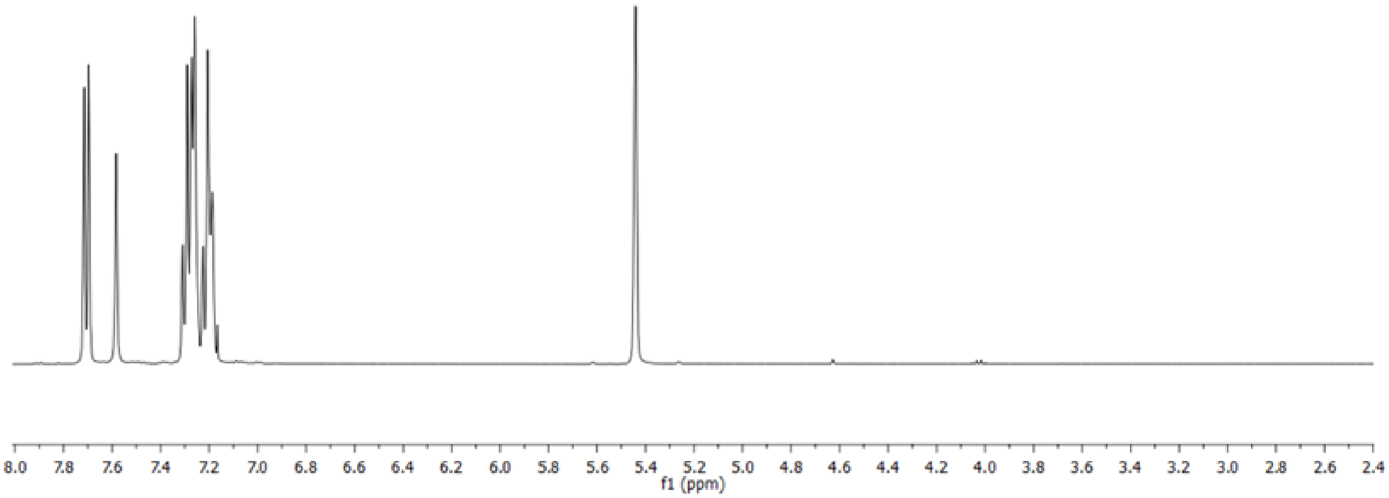

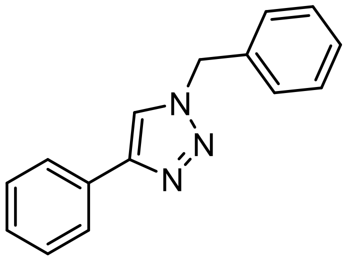
N
N
N

1

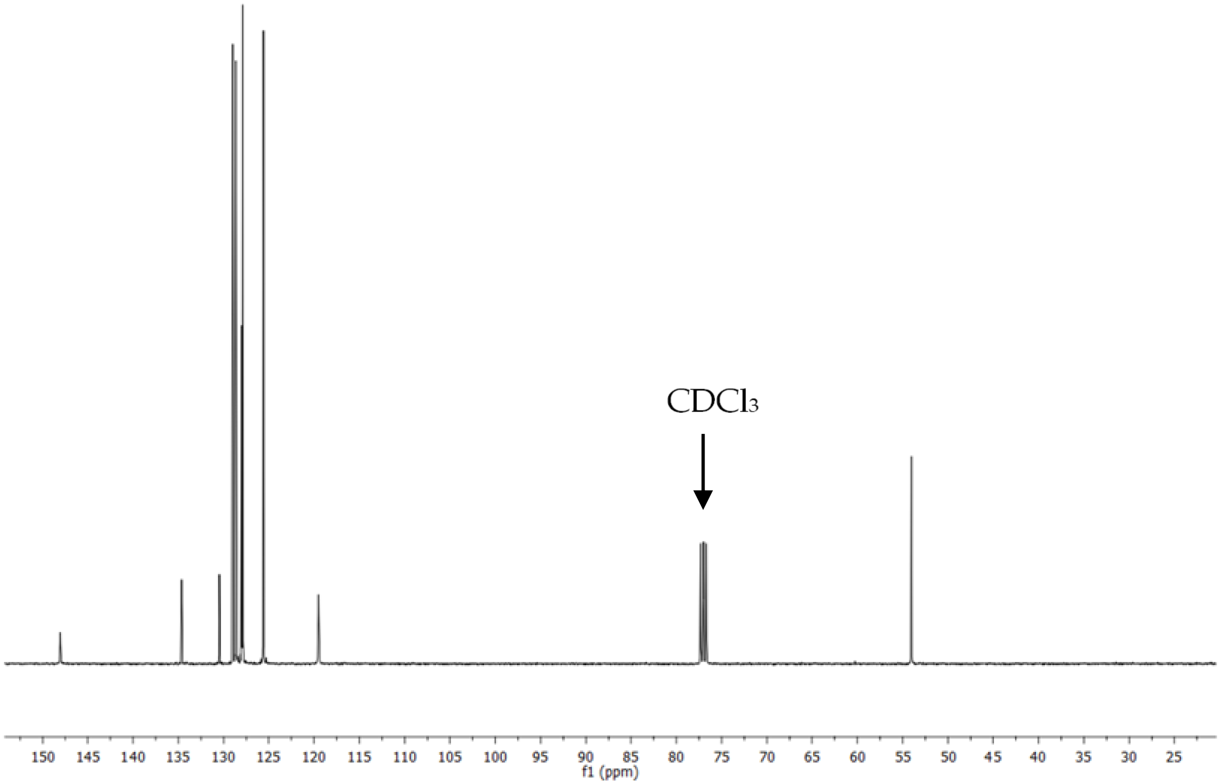
CDCl3
150 145 140 135 130 125 120 115 110 105 100 95 90 85 80 75 70 65 60 55 50 45 40 35 30 25
f1 (ppm)

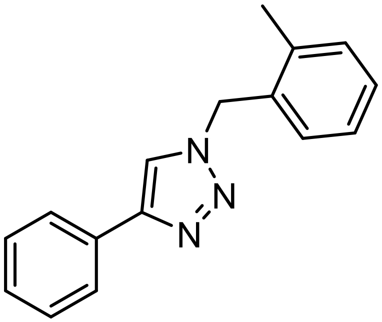

2

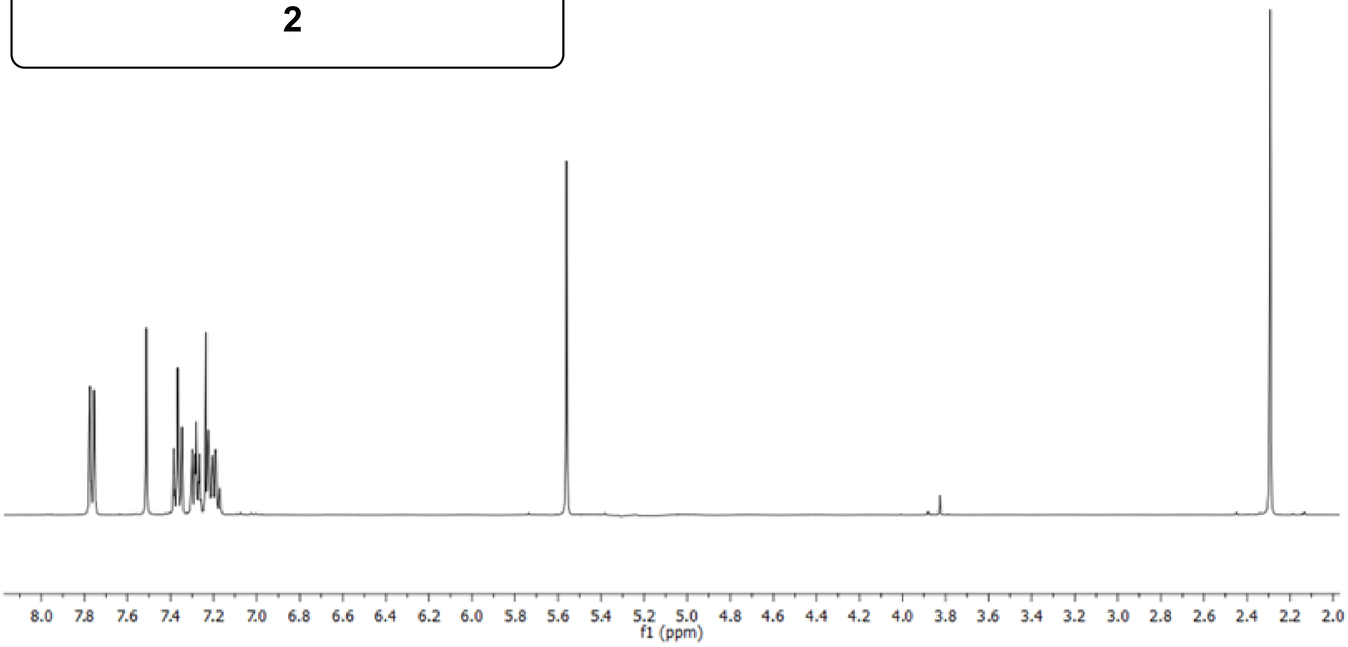
8.0 7.8 7.6 7.4 7.2 7.0 6.8 6.6 6.4 6.2 6.0 5.8 5.6 5.4 5.2 5.0 4.8 4.6 4.4 4.2 4.0 3.8 3.6 3.4 3.2 3.0 2.8 2.6 2.4 2.2 2.0
f1 (ppm)

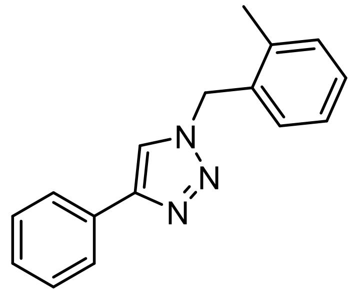

2

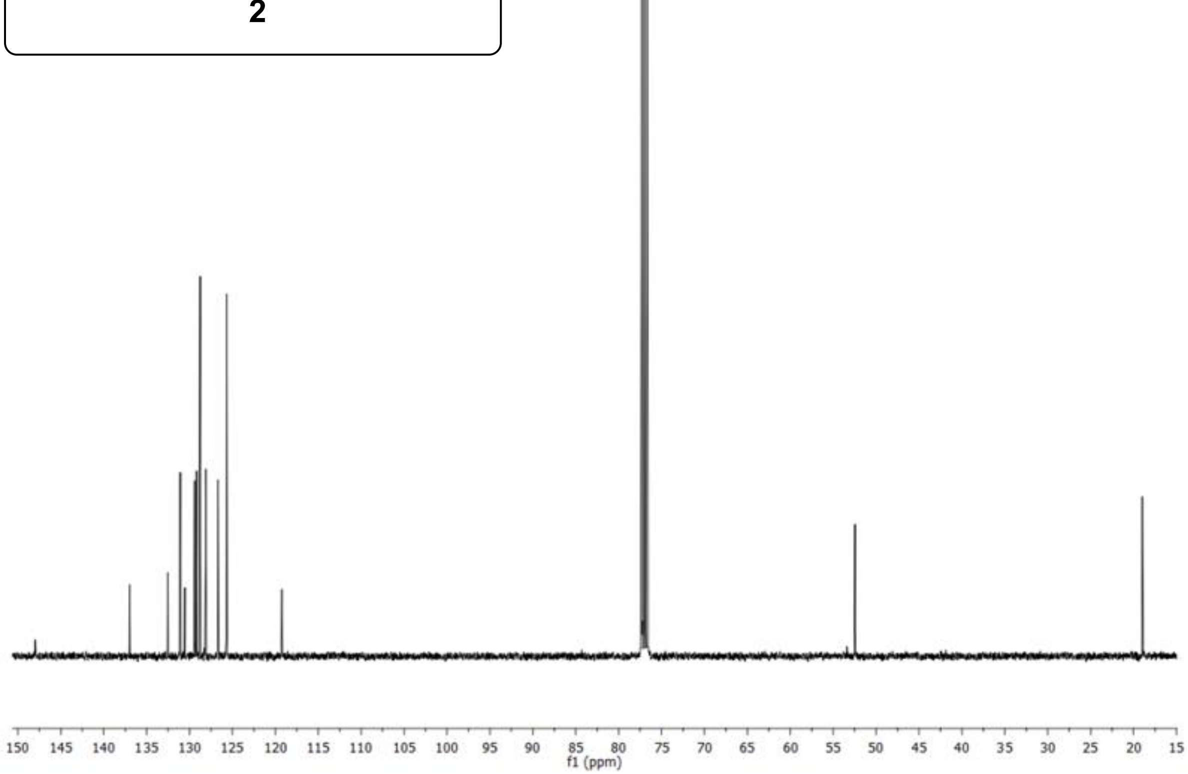
CDCl3
150 145 140 135 130 125 120 115 110 105 100 95 90 85 80 75 70 65 60 55 50 45 40 35 30 25 20 15
f1 (ppm)

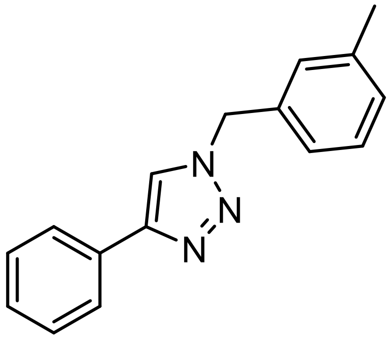

3

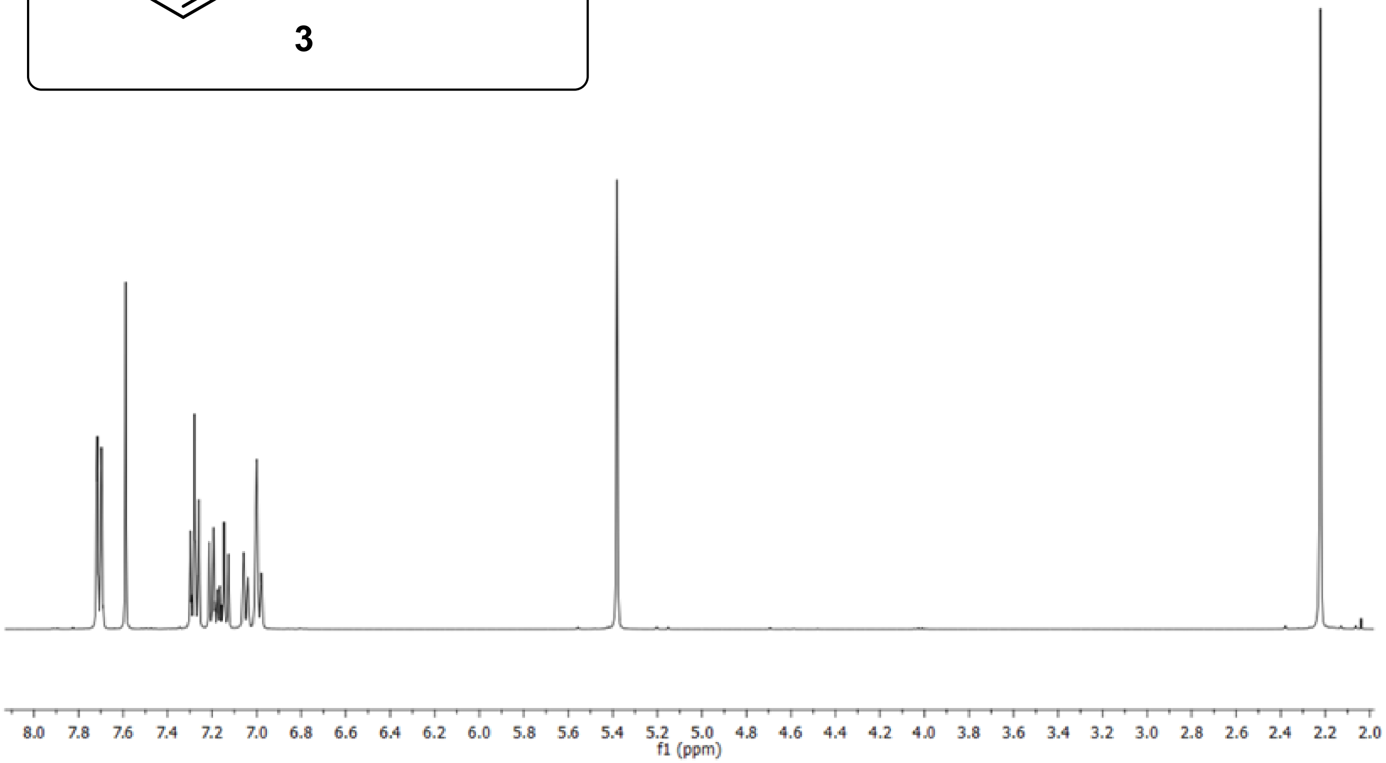
8.0 7.8 7.6 7.4 7.2 7.0 6.8 6.6 6.4 6.2 6.0 5.8 5.6 5.4 5.2 5.0 4.8 4.6 4.4 4.2 4.0 3.8 3.6 3.4 3.2 3.0 2.8 2.6 2.4 2.2 2.0
f1 (ppm)

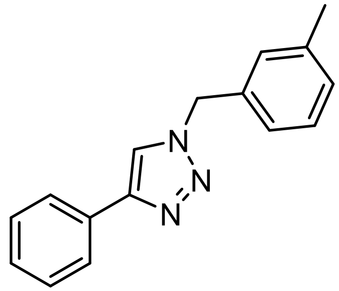

3

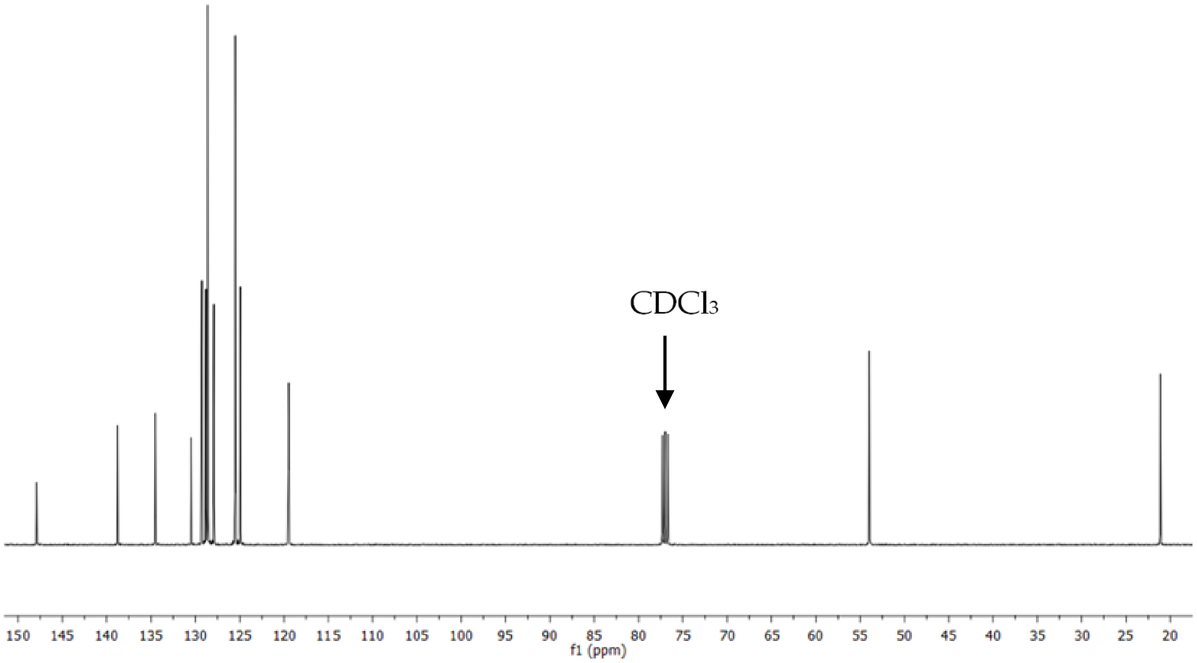
CDCl3
150 145 140 135 130 125 120 115 110 105 100 95 90 85 80 75 70 65 60 55 50 45 40 35 30 25 20
f1 (ppm)

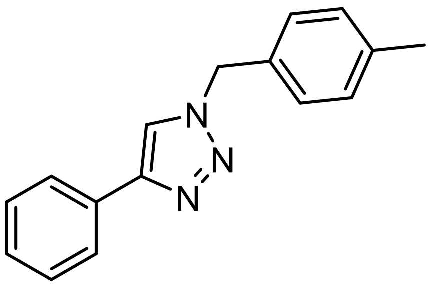

4

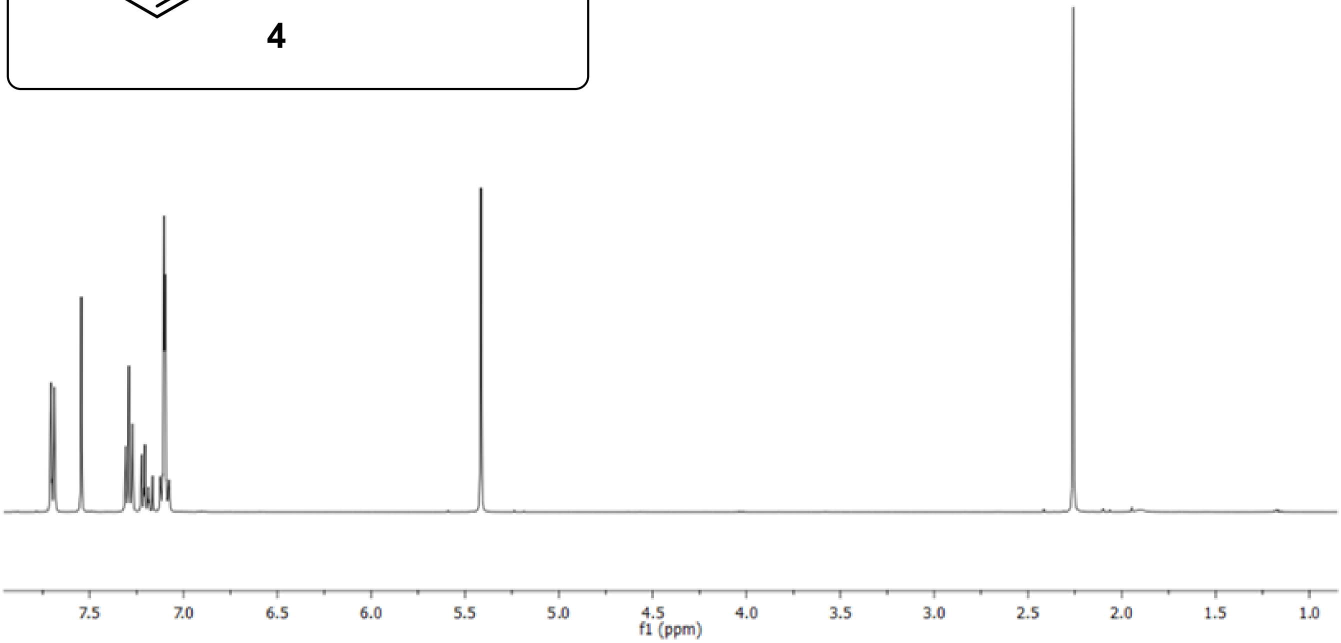

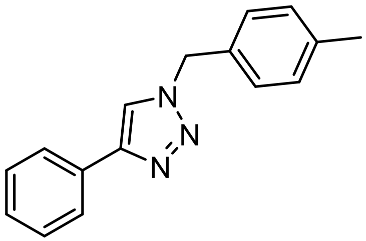

4

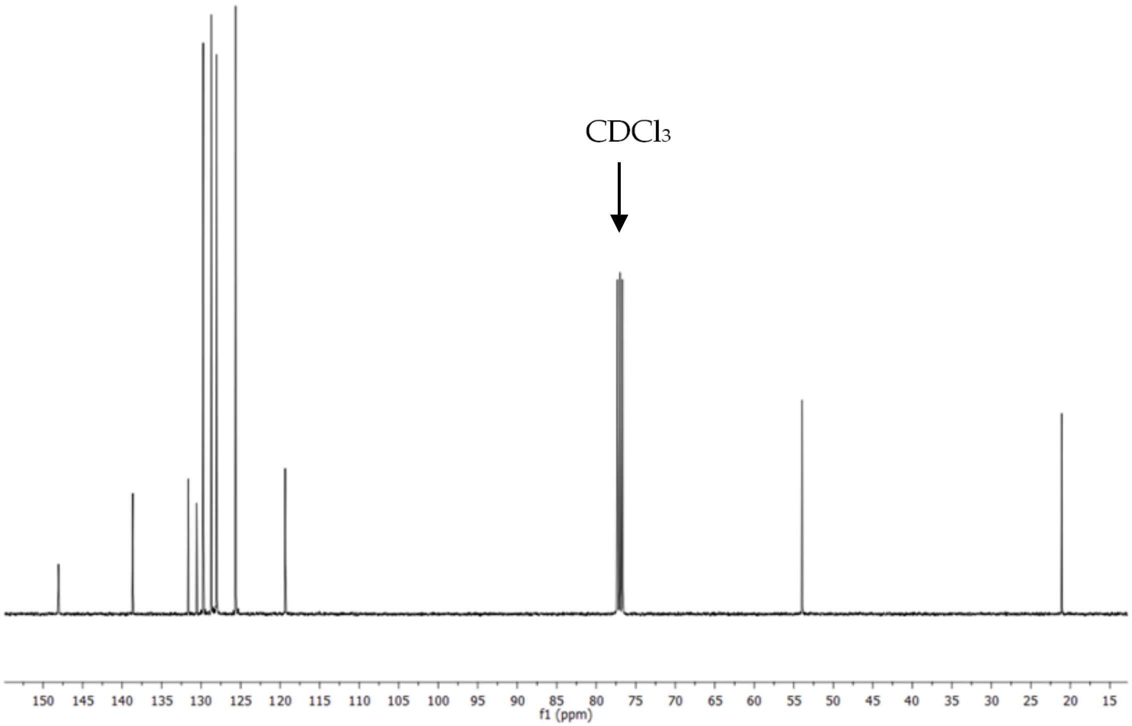
CDCl3
150 145 140 135 130 125 120 115 110 105 100 95 90 85 80 75 70 65 60 55 50 45 40 35 30 25 20 15
f1 (ppm)

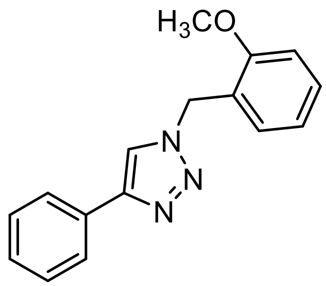

5

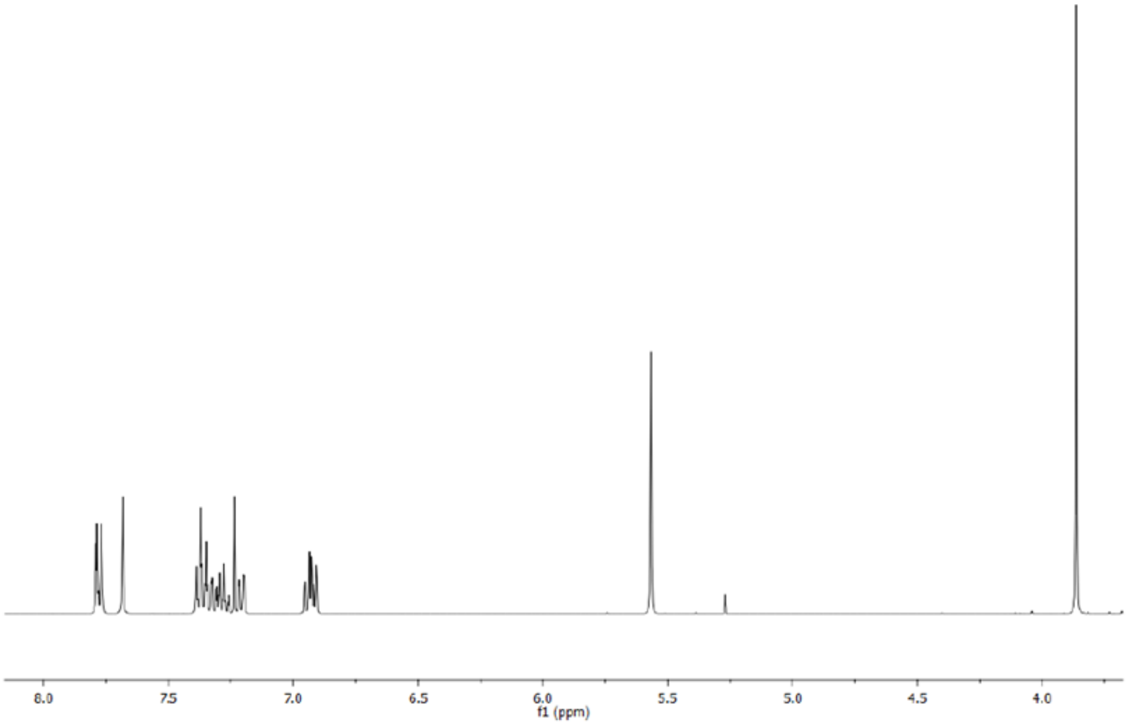

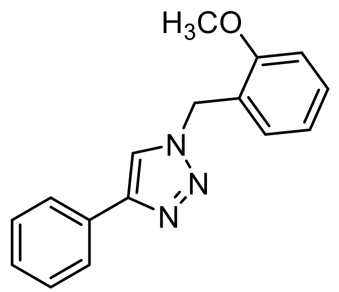
H3CO
N
N
N

5

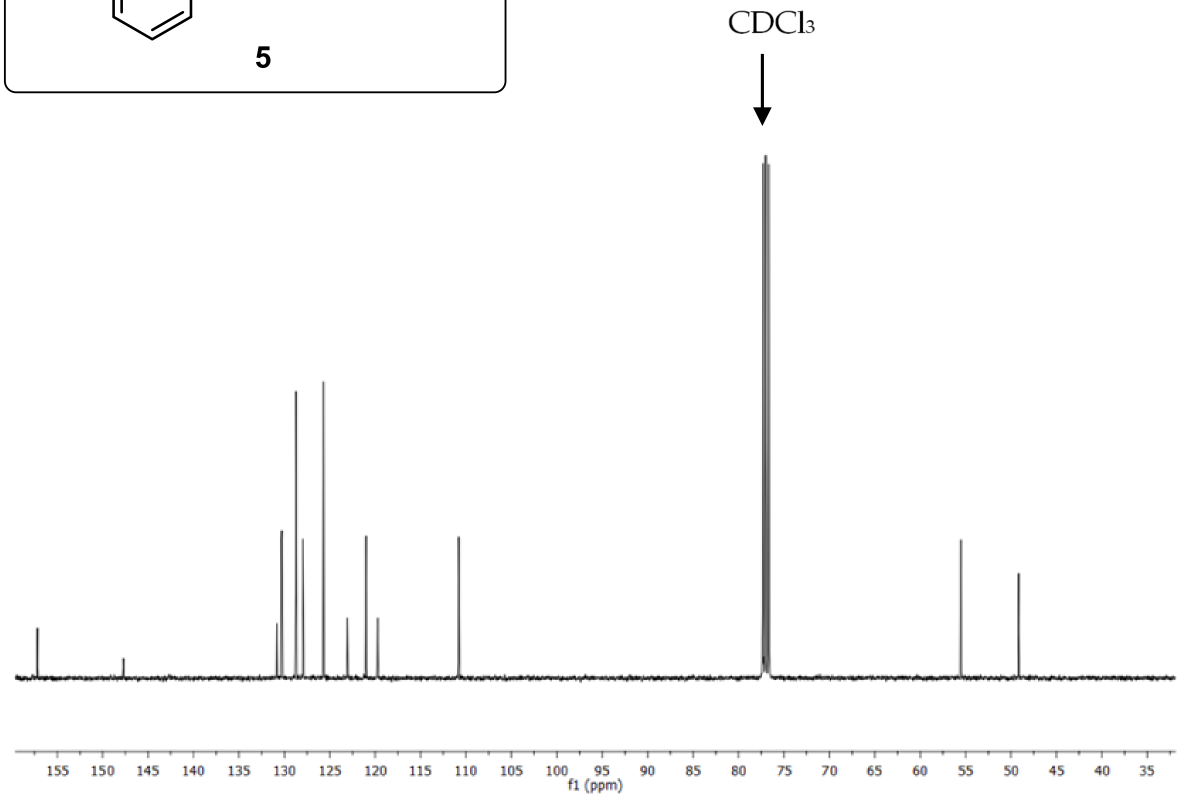
CDCl3
155
150
145
140
135
130
125
120
115
110
105
100
95
90
85
80
75
70
65
60
55
50
45
40
35
f1 (ppm)

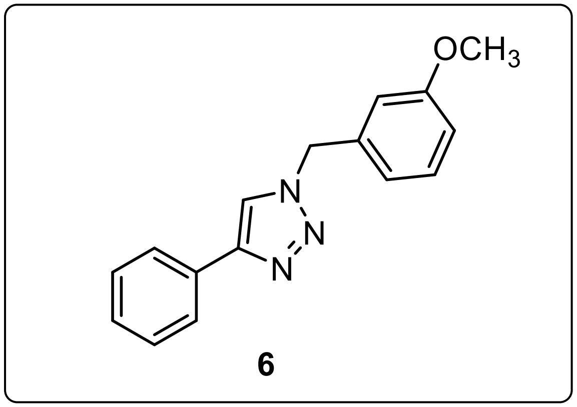
OCH3
N
N
N
6

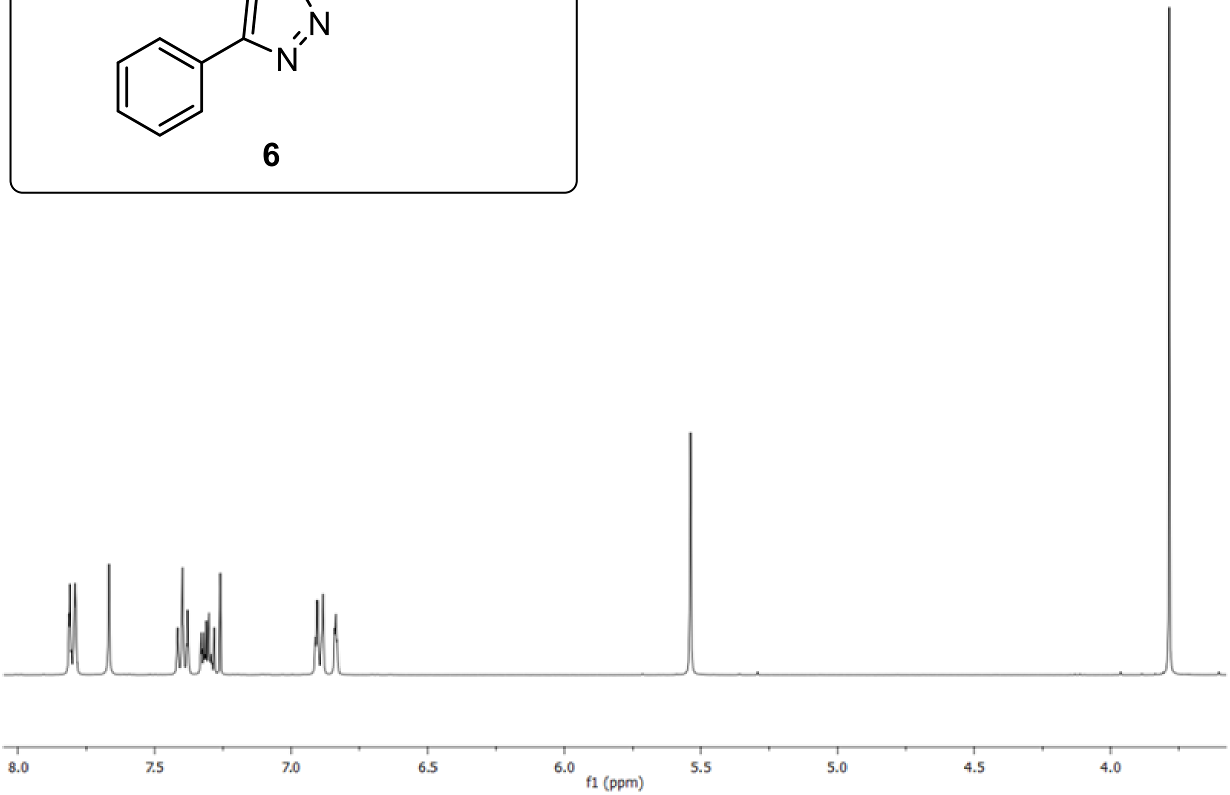
8.0
7.5
7.0
6.5
6.0
5.5
5.0
4.5
4.0
f1 (ppm)

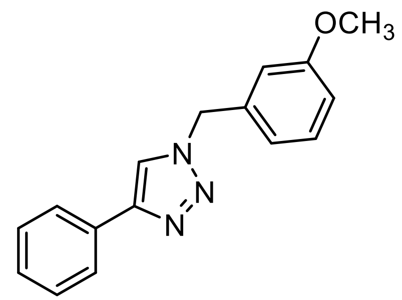
OCH3
N
N
N

6

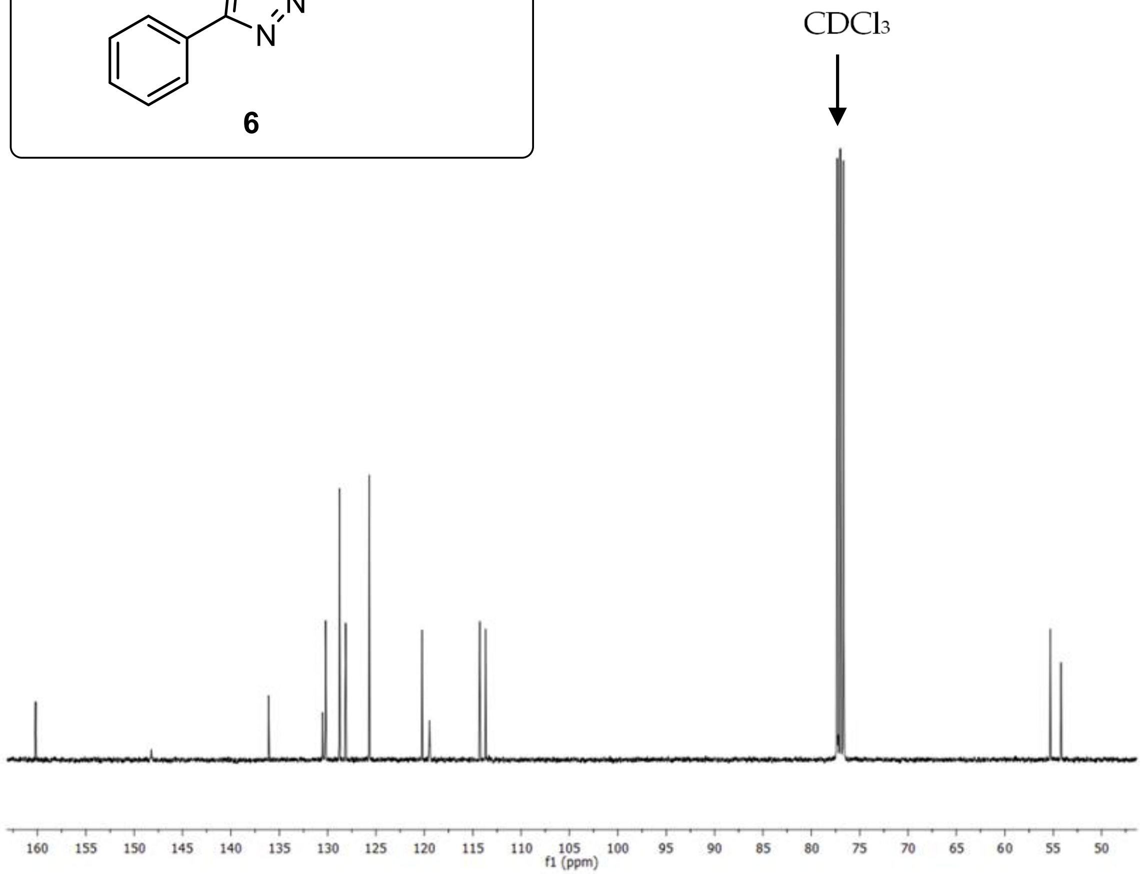
CDCl3
160 155 150 145 140 135 130 125 120 115 110 105 100 95 90 85 80 75 70 65 60 55 50
f1 (ppm)

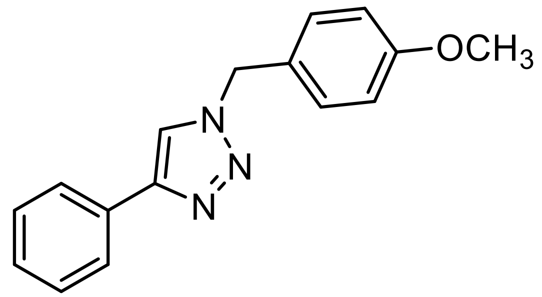

N
N
N
OCH3

7

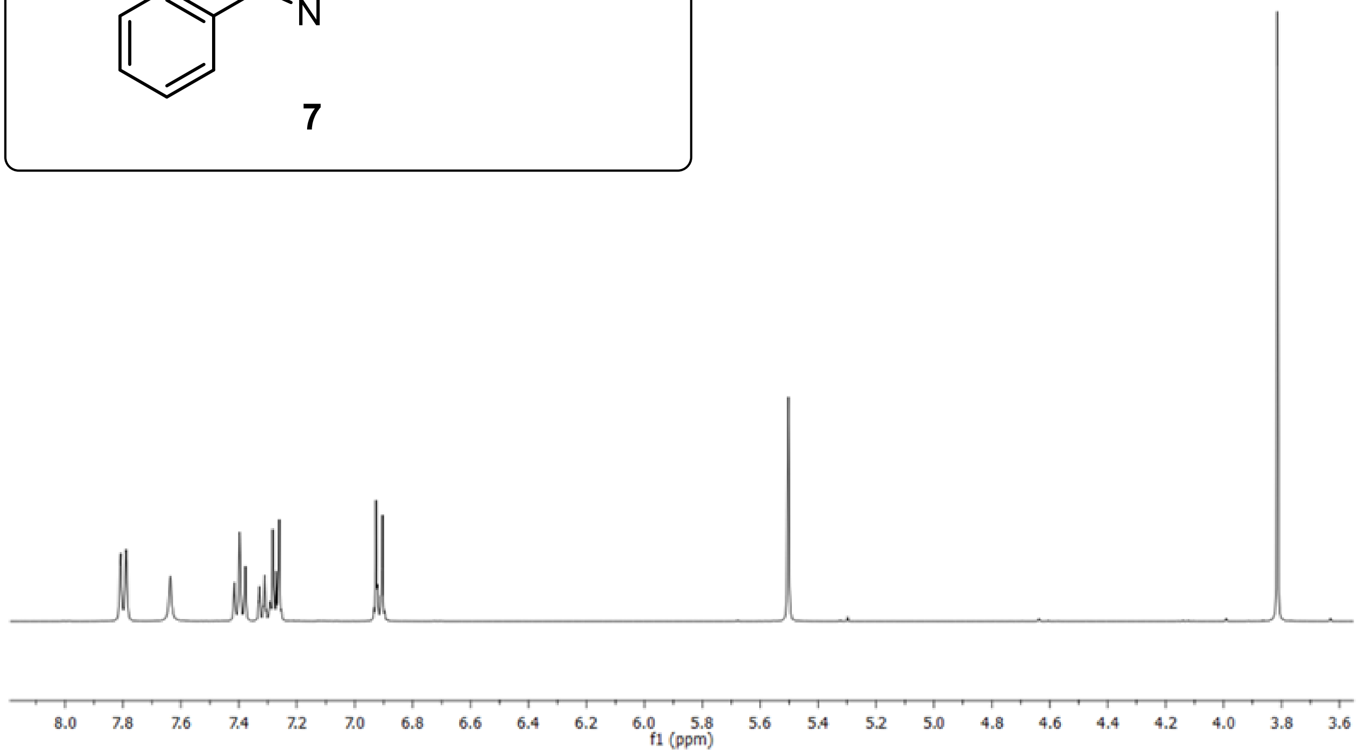

8.0
7.8
7.6
7.4
7.2
7.0
6.8
6.6
6.4
6.2
6.0
5.8
5.6
5.4
5.2
5.0
4.8
4.6
4.4
4.2
4.0
3.8
3.6
f1 (ppm)

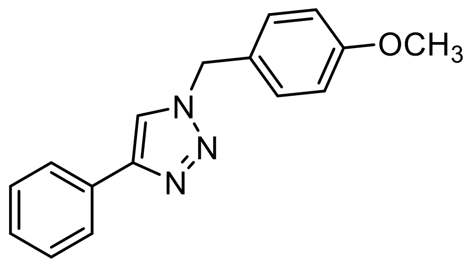
OCH3
N
N
N

7

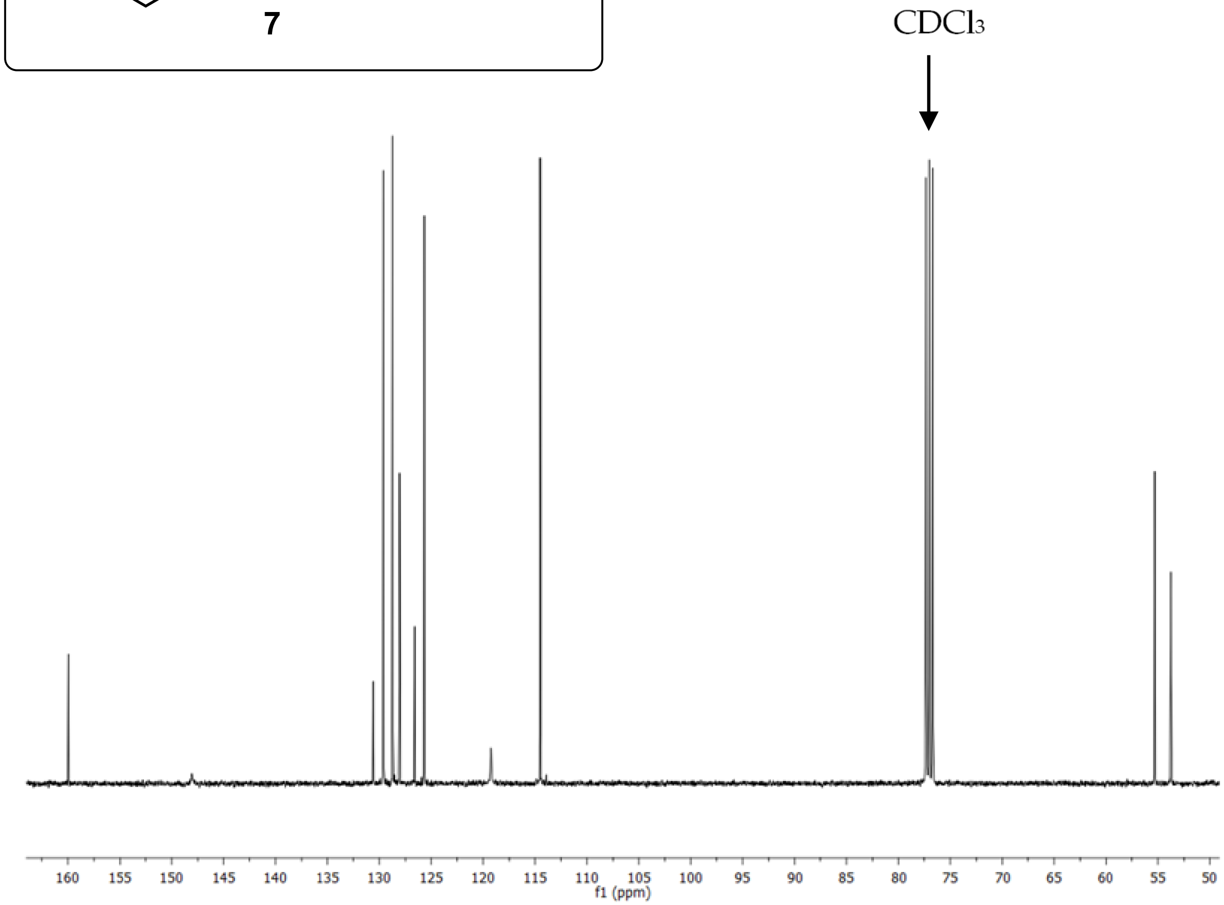
CDCl3
160 155 150 145 140 135 130 125 120 115 110 105 100 95 90 85 80 75 70 65 60 55 50
f1 (ppm)

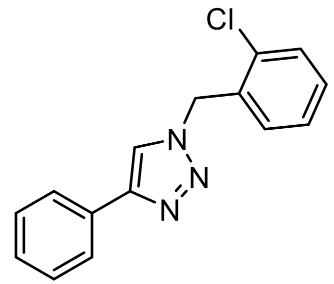
Cl
N
N
N

8

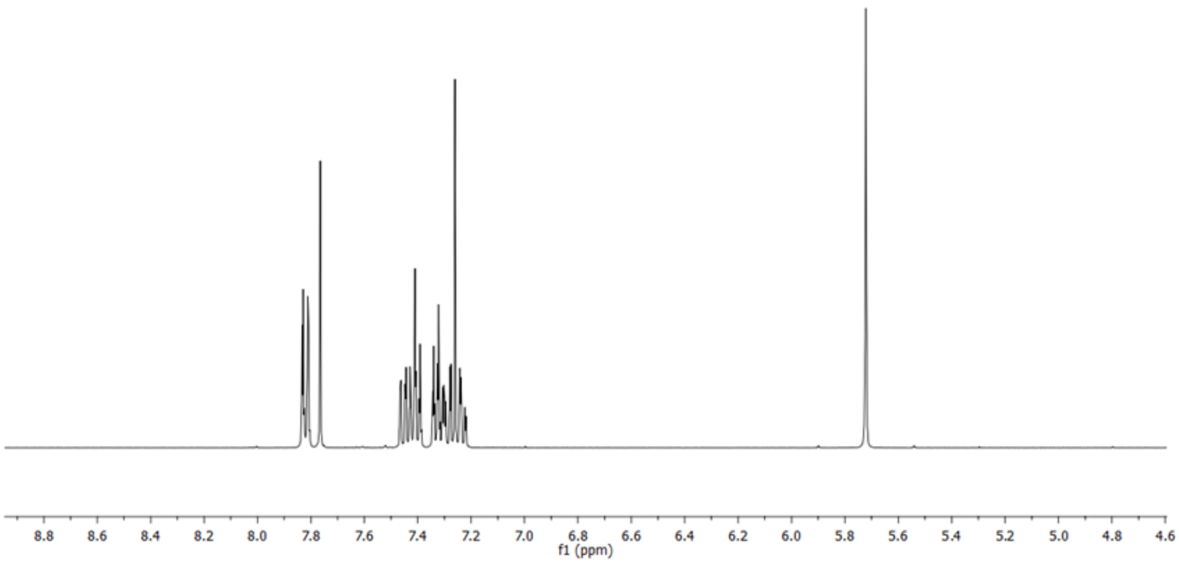
8.8
8.6
8.4
8.2
8.0
7.8
7.6
7.4
7.2
7.0
6.8
6.6
6.4
6.2
6.0
5.8
5.6
5.4
5.2
5.0
4.8
4.6
f1 (ppm)

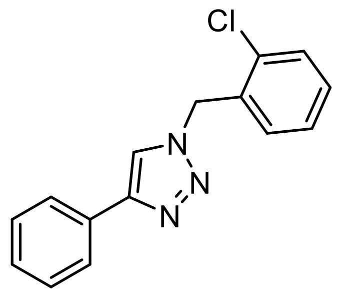
Cl
N
N
N

8

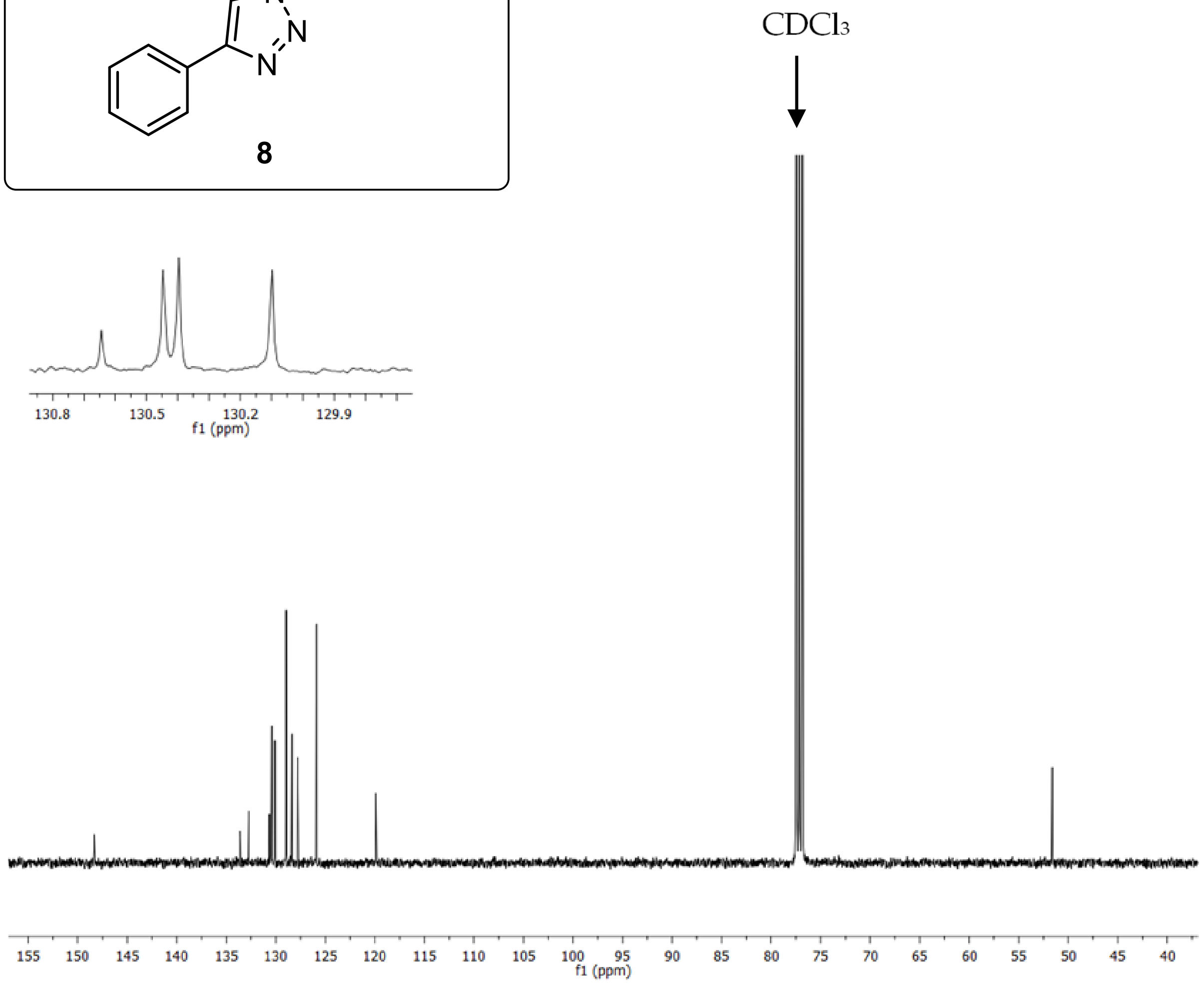
CDCl3
130.8
130.5
130.2
129.9
f1 (ppm)
155
150
145
140
135
130
125
120
115
110
105
100
95
90
85
80
75
70
65
60
55
50
45
40
f1 (ppm)

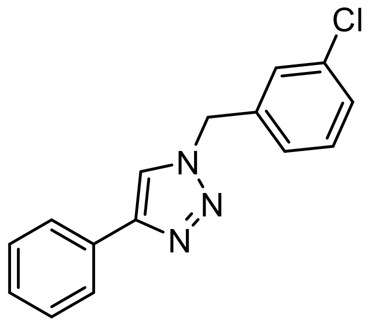

9

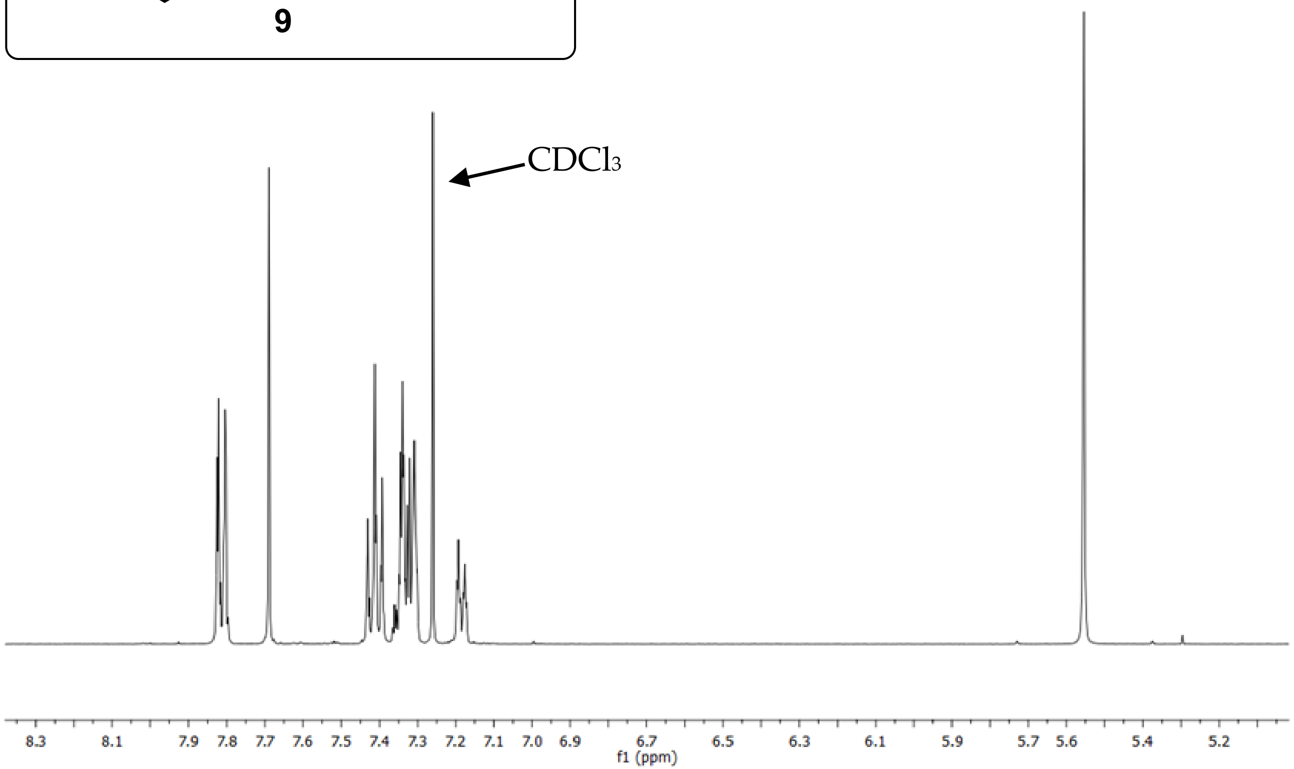
CDCl3
8.3
8.1
7.9
7.8
7.7
7.6
7.5
7.4
7.3
7.2
7.1
7.0
6.9
6.7
6.5
6.3
6.1
5.9
5.7
5.6
5.4
5.2
f1 (ppm)

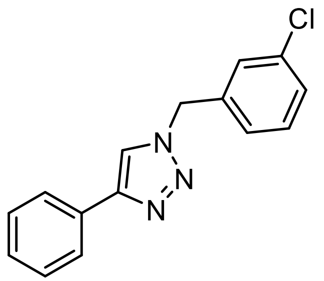

9

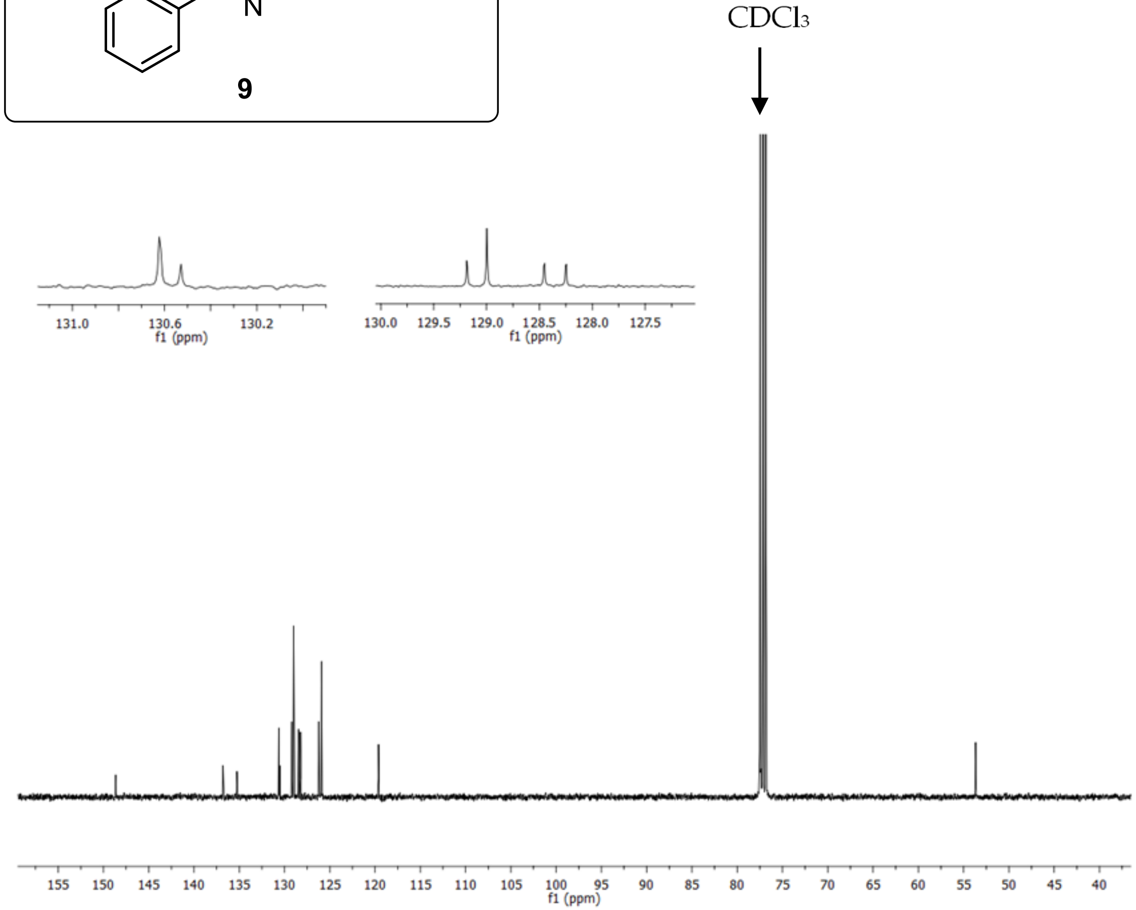

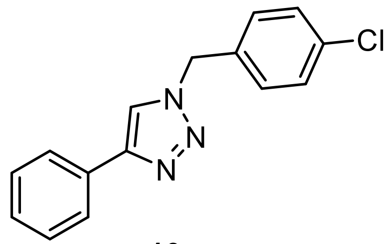

Cl
N
N
N

10

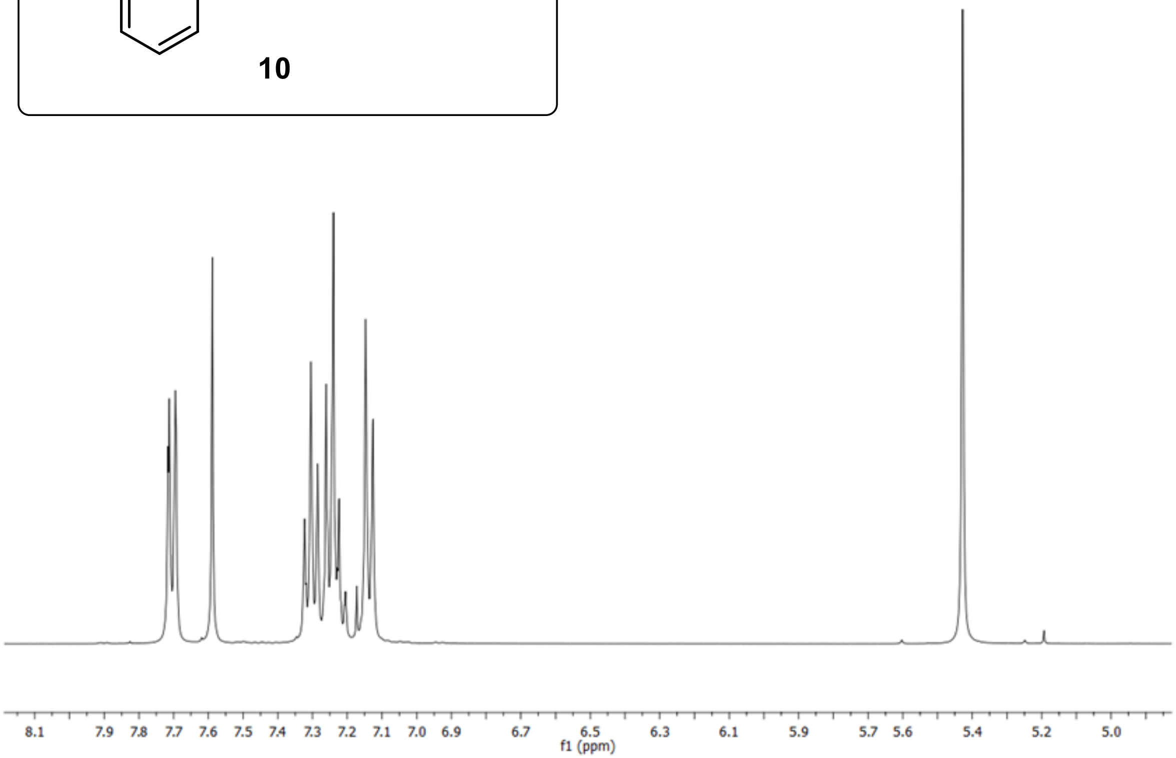

8.1
7.9
7.8
7.7
7.6
7.5
7.4
7.3
7.2
7.1
7.0
6.9
6.7
6.5
6.3
6.1
5.9
5.7
5.6
5.4
5.2
5.0
f1 (ppm)

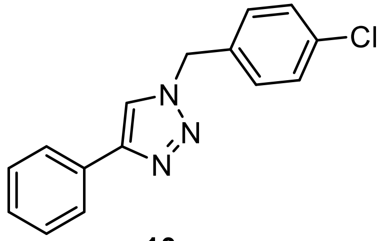
Cl
N
N
N

10

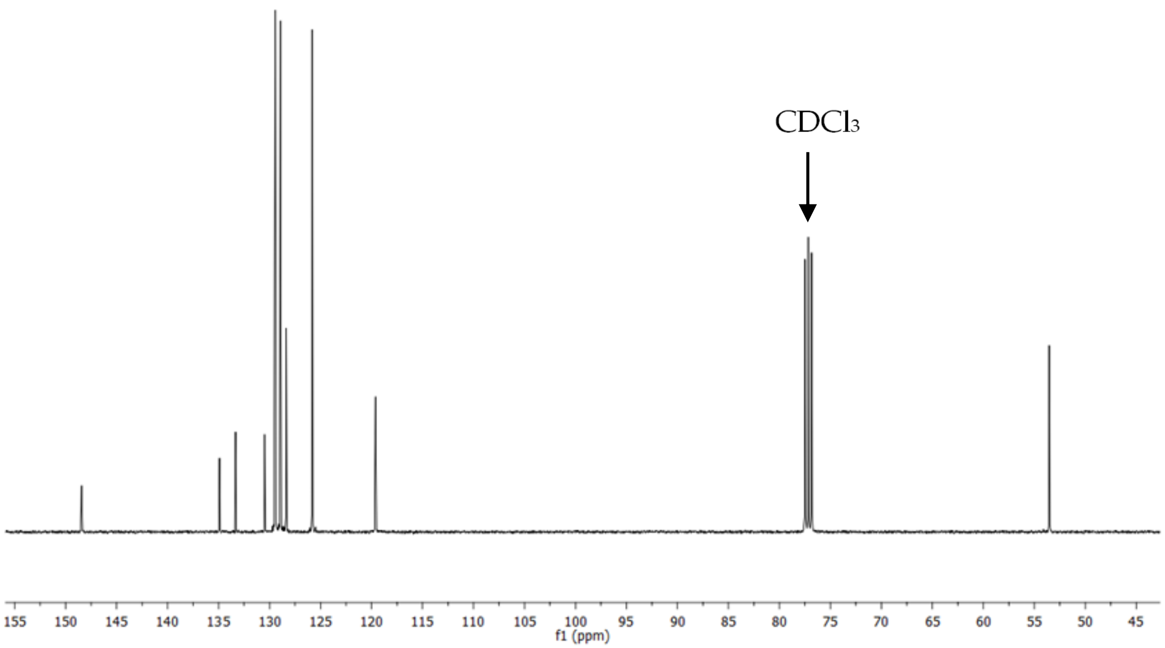
CDCl3
155
150
145
140
135
130
125
120
115
110
105
100
95
90
85
80
75
70
65
60
55
50
45
f1 (ppm)

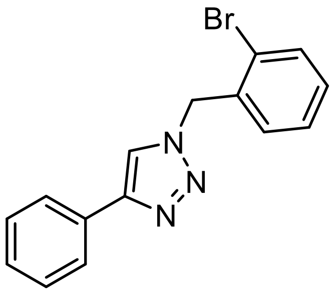

11

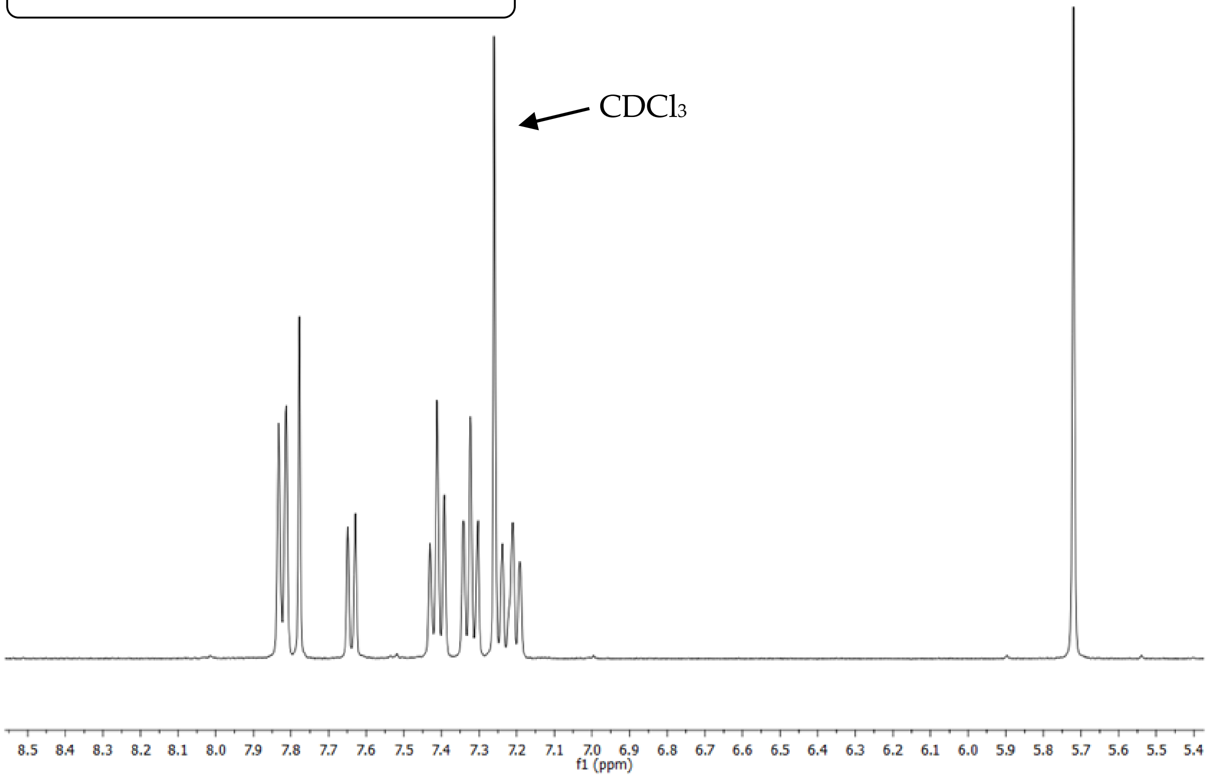

CDCl3
8.5 8.4 8.3 8.2 8.1 8.0 7.9 7.8 7.7 7.6 7.5 7.4 7.3 7.2 7.1 7.0 6.9 6.8 6.7 6.6 6.5 6.4 6.3 6.2 6.1 6.0 5.9 5.8 5.7 5.6 5.5 5.4
f1 (ppm)

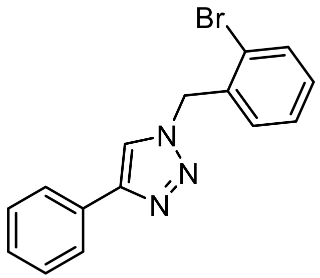
Br
N
N
N

11

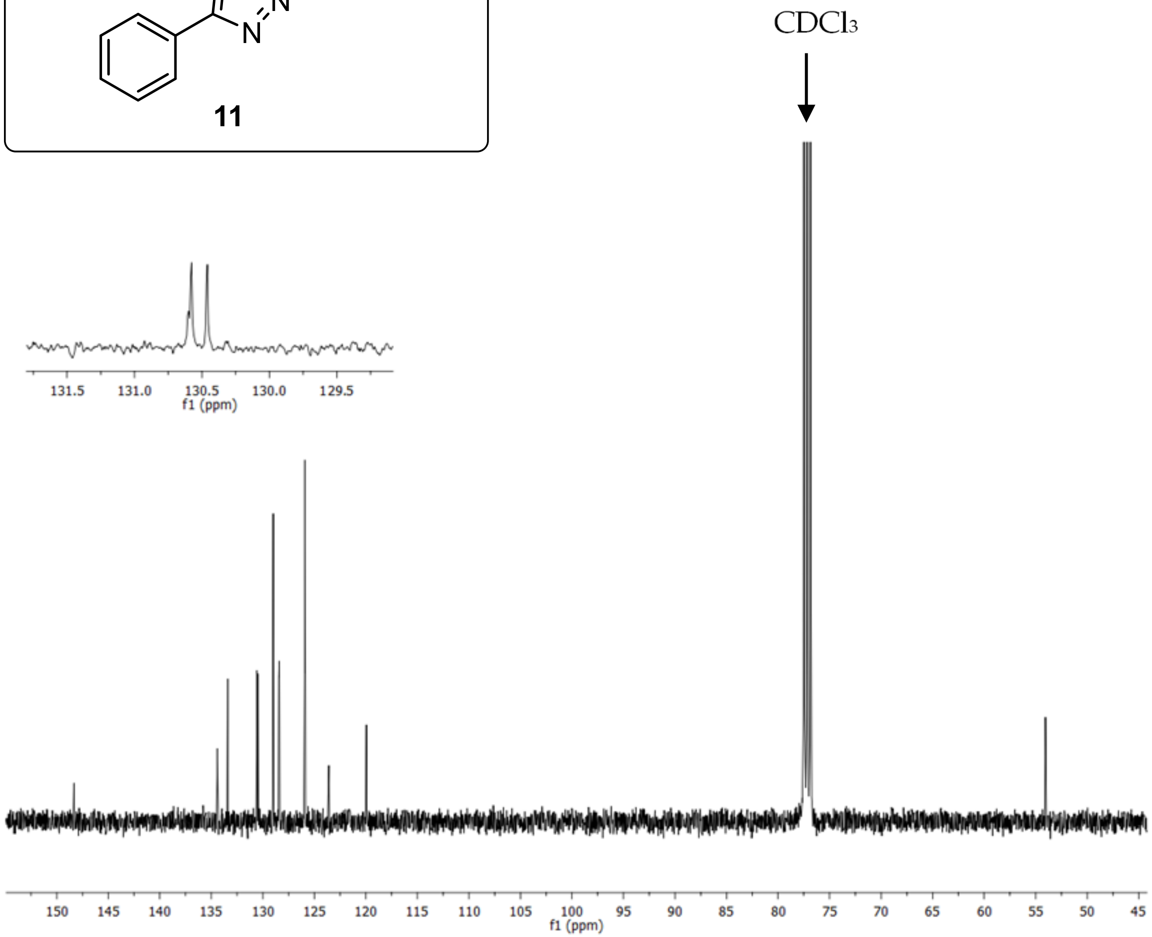
CDCl3
131.5 131.0 130.5 130.0 129.5
f1 (ppm)
150 145 140 135 130 125 120 115 110 105 100 95 90 85 80 75 70 65 60 55 50 45
f1 (ppm)

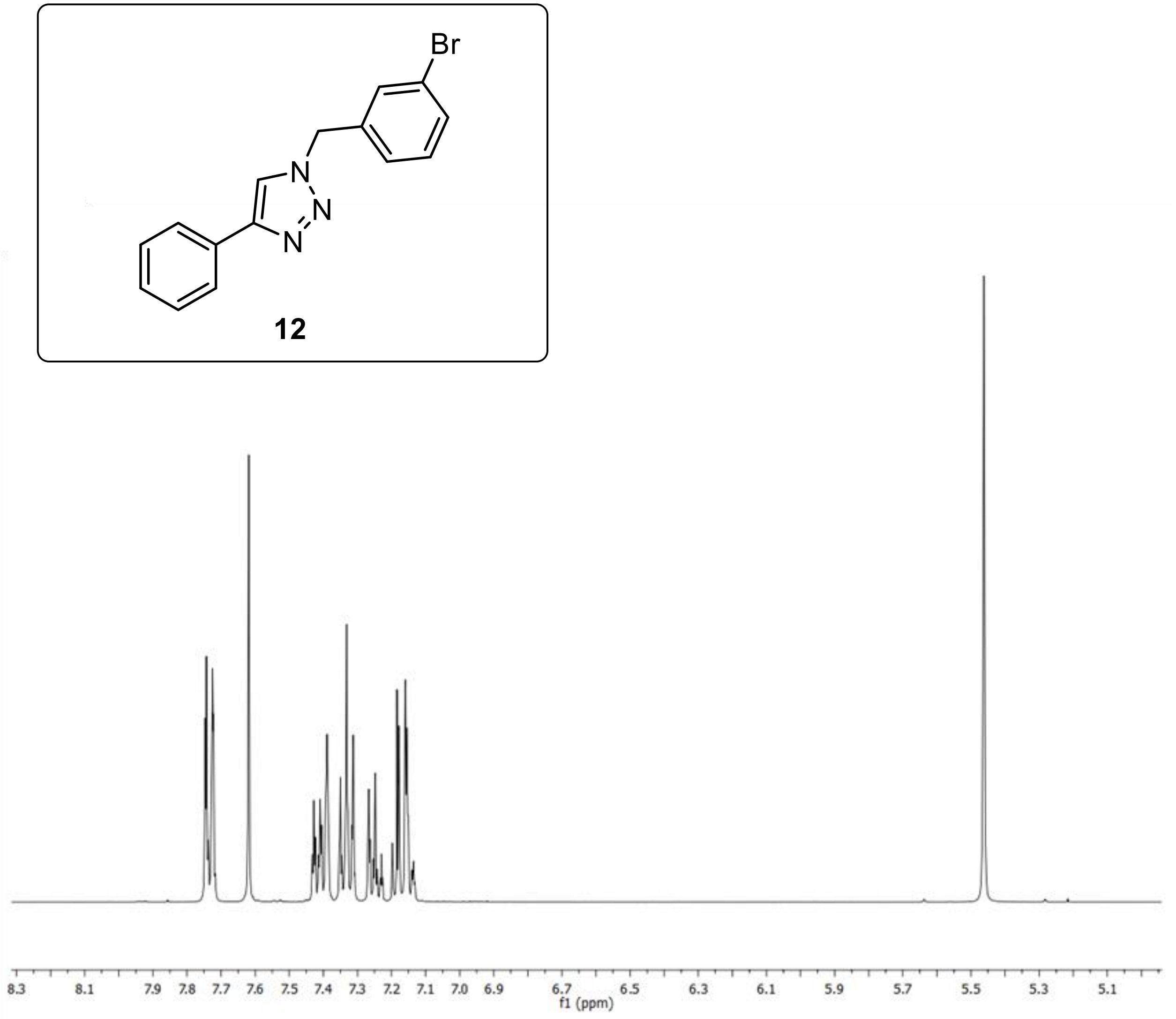
Br
N
N
N
12
8.3 8.1 7.9 7.8 7.7 7.6 7.5 7.4 7.3 7.2 7.1 7.0 6.9 6.7 6.5 6.3 6.1 5.9 5.7 5.5 5.3 5.1
f1 (ppm)

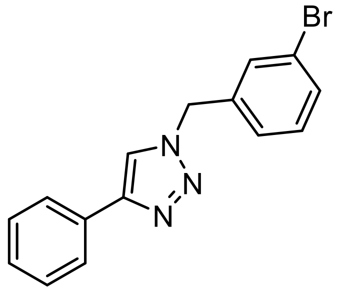
Br
N
N
N

12

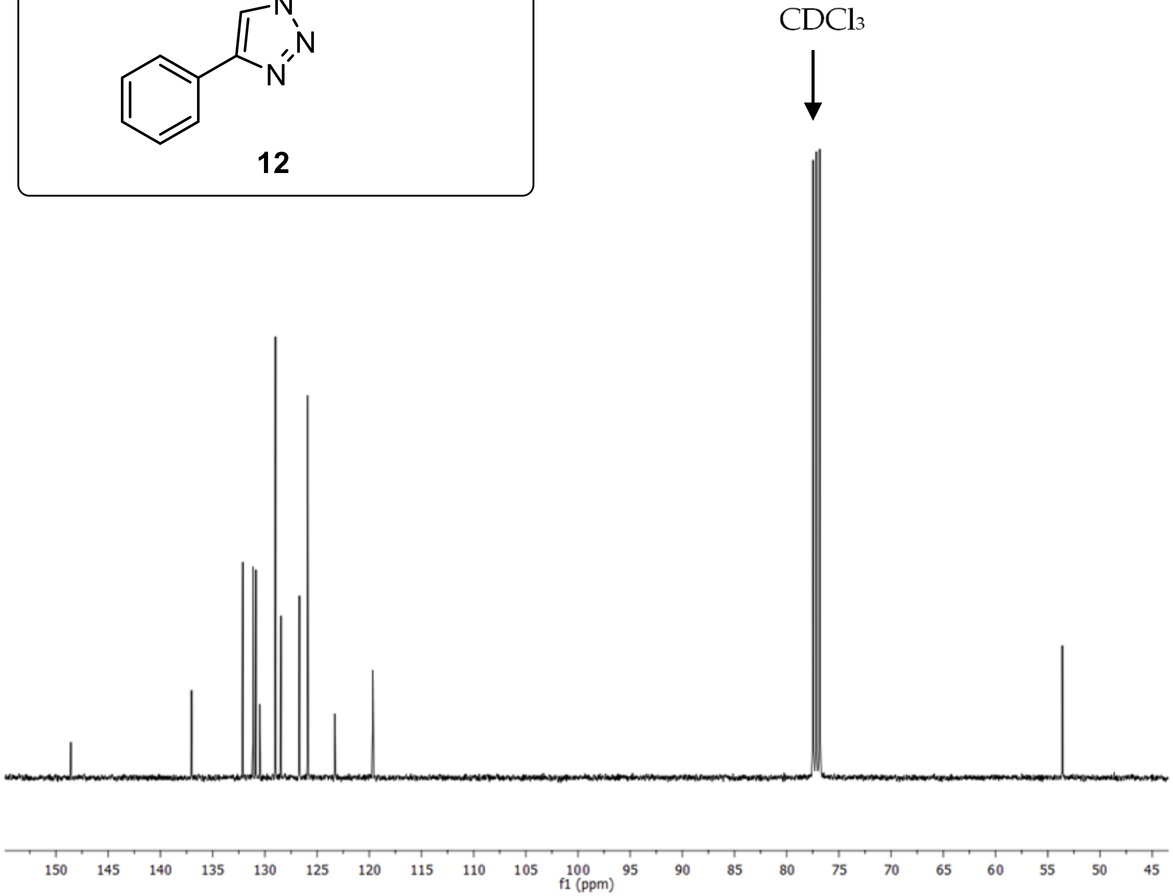
CDCl3
150 145 140 135 130 125 120 115 110 105 100 95 90 85 80 75 70 65 60 55 50 45
f1 (ppm)

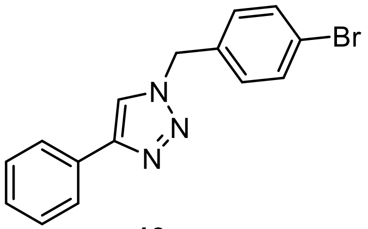
N
N
N
Br

13

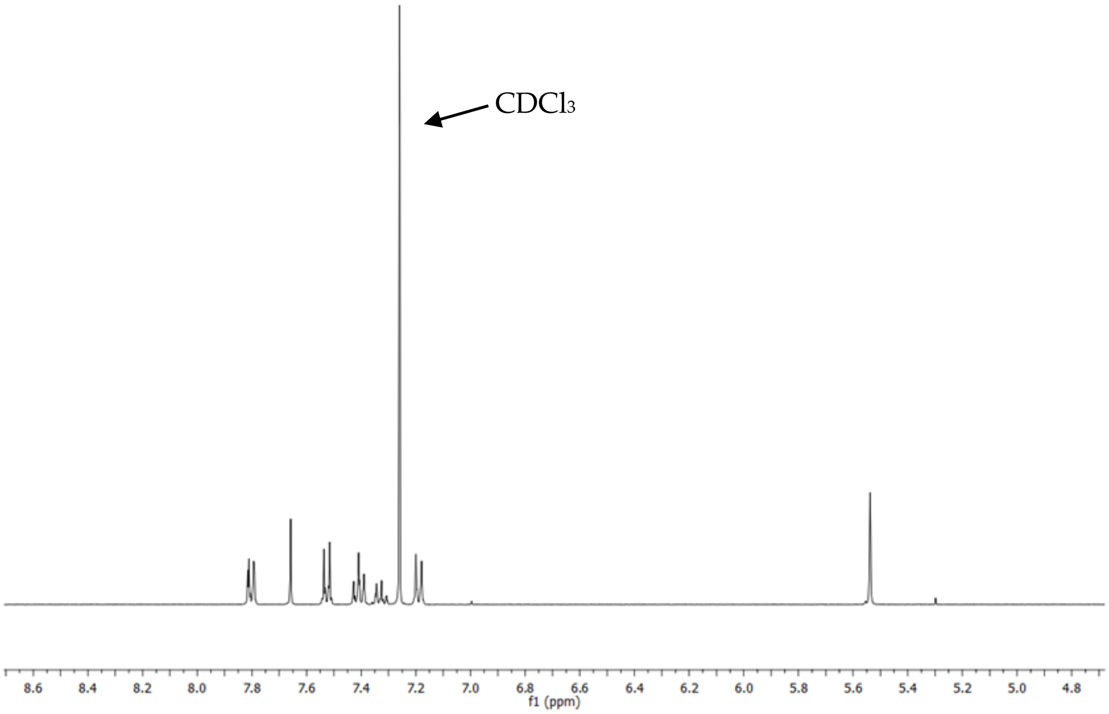
CDCl3
8.6 8.4 8.2 8.0 7.8 7.6 7.4 7.2 7.0 6.8 6.6 6.4 6.2 6.0 5.8 5.6 5.4 5.2 5.0 4.8
f1 (ppm)

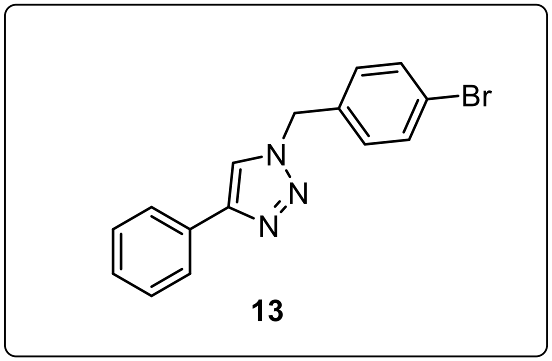
Br
N
N
N
13

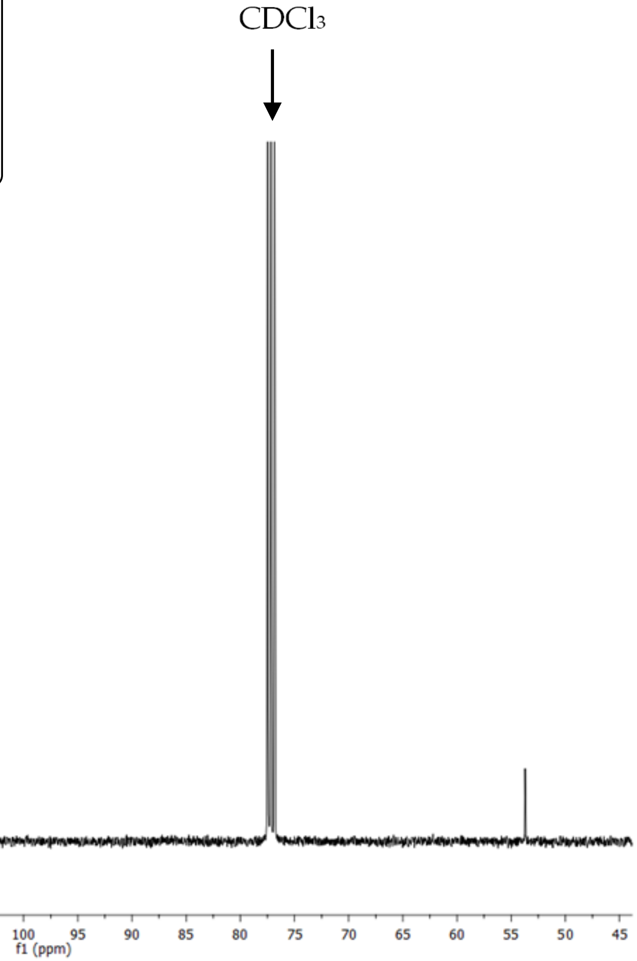
CDCl3
150 145 140 135 130 125 120 115 110 105 100 95 90 85 80 75 70 65 60 55 50 45
f1 (ppm)

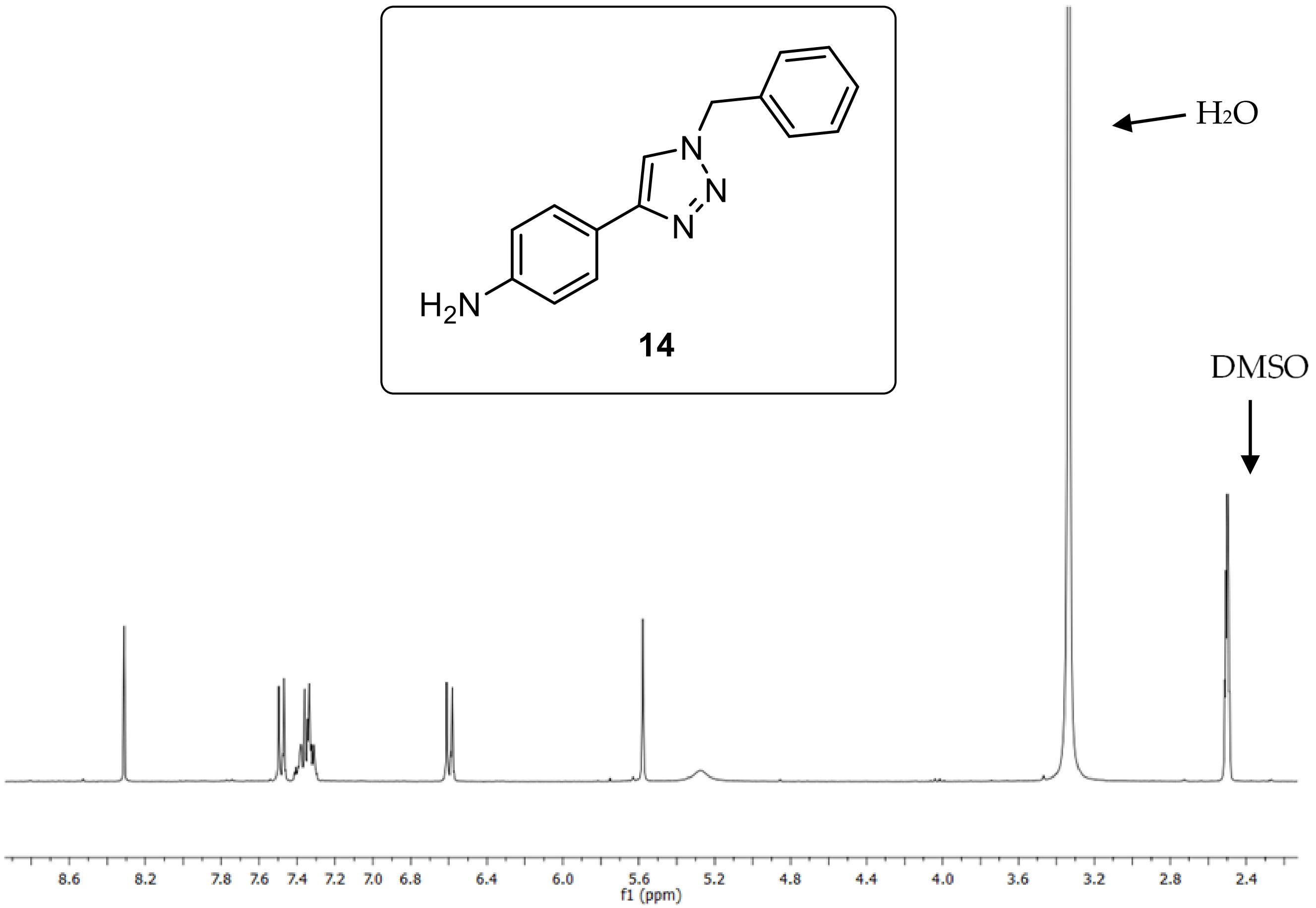
H2N
N
N
N
14
H2O
DMSO
8.6 8.2 7.8 7.6 7.4 7.2 7.0 6.8 6.4 6.0 5.6 5.2 4.8 4.4 4.0 3.6 3.2 2.8 2.4
f1 (ppm)

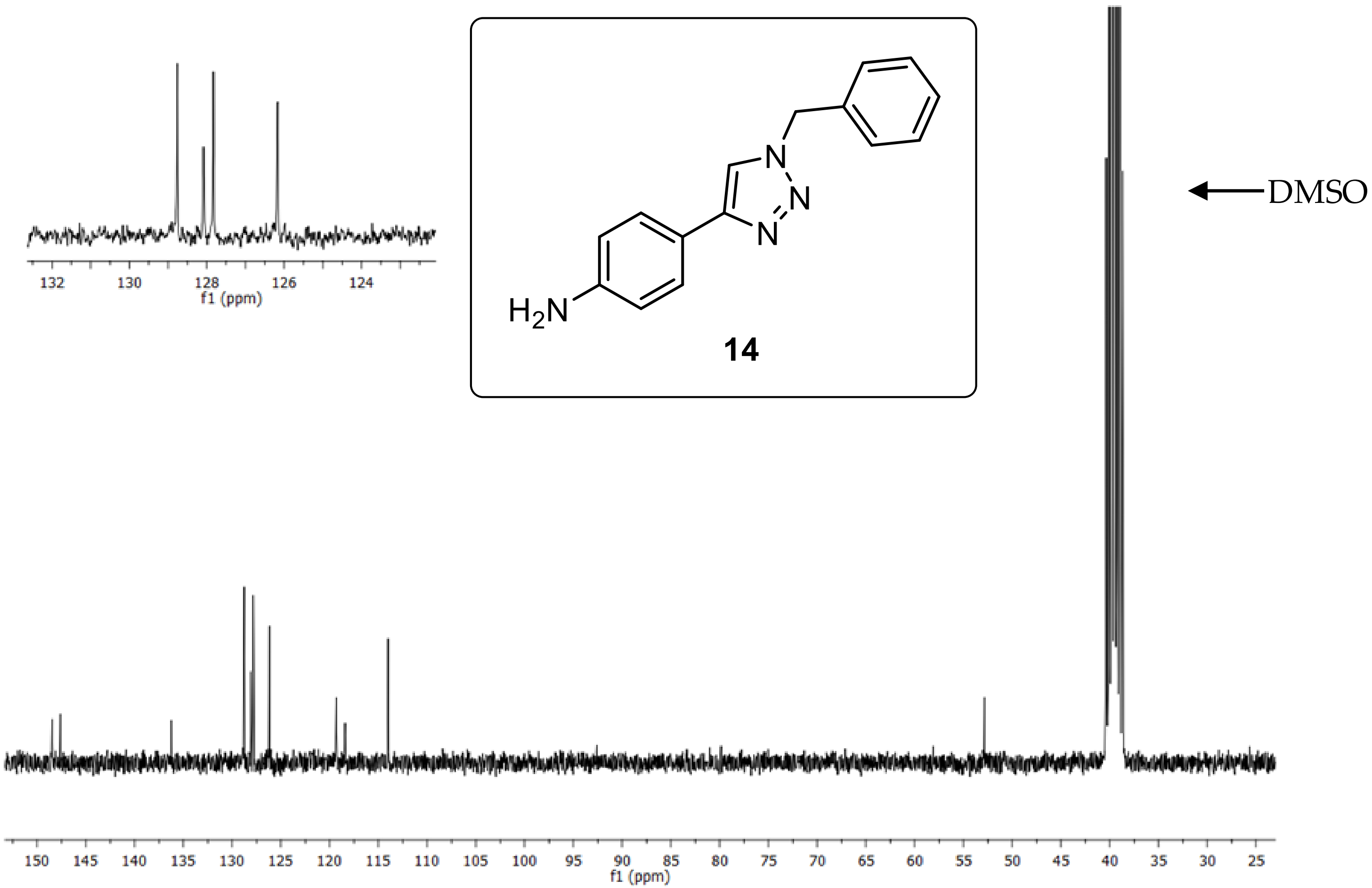
14
DMSO
f1 (ppm)
f1 (ppm)

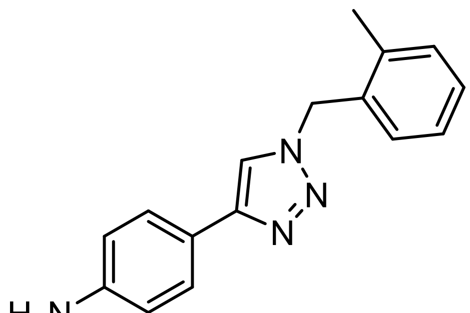

15

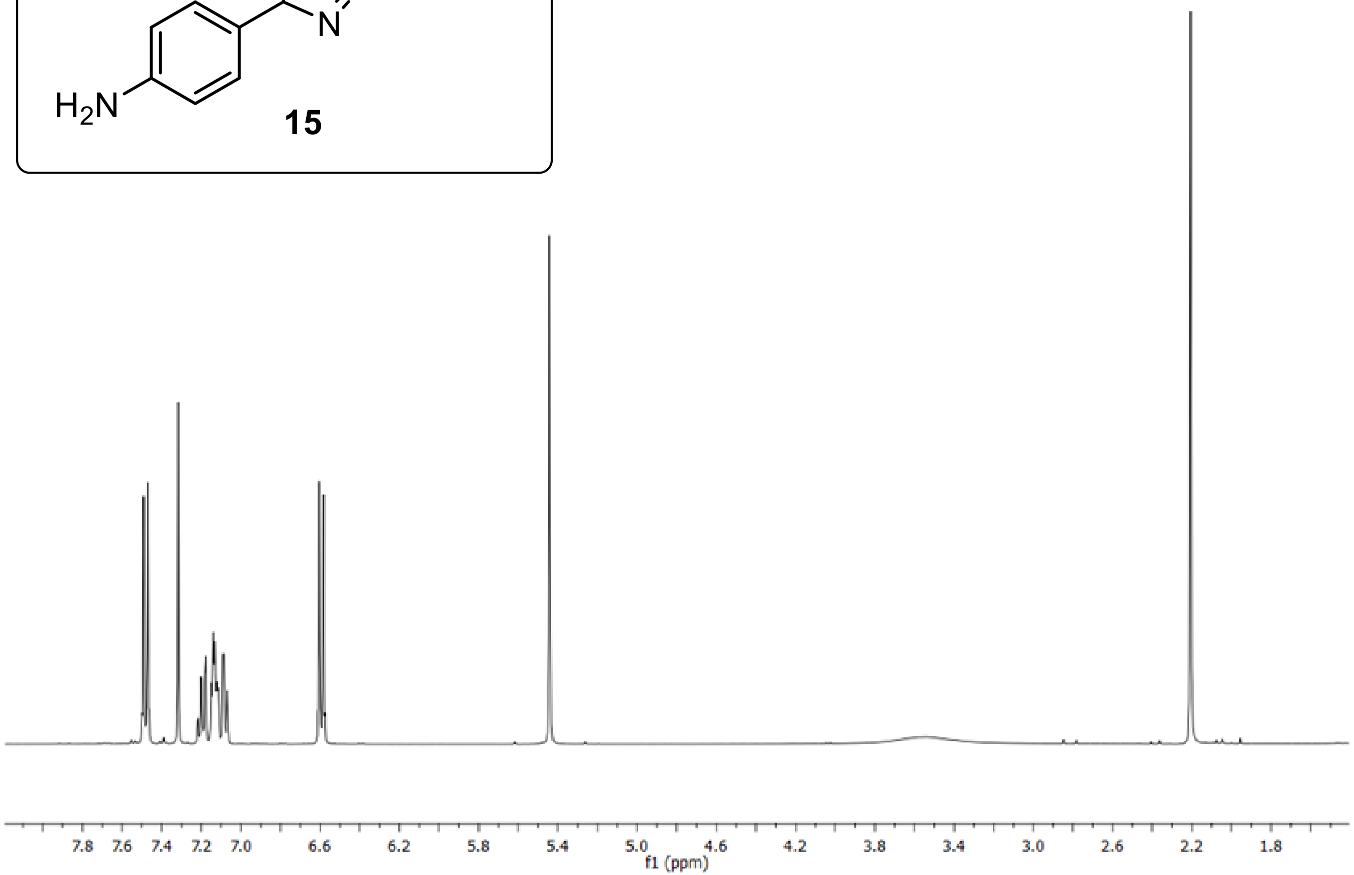

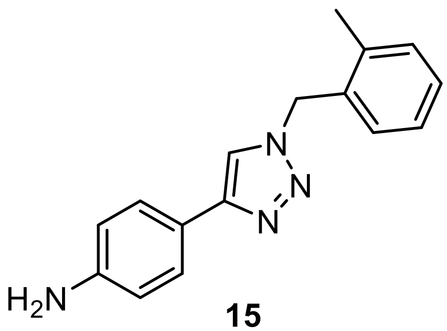
H2N
N
N
N

15

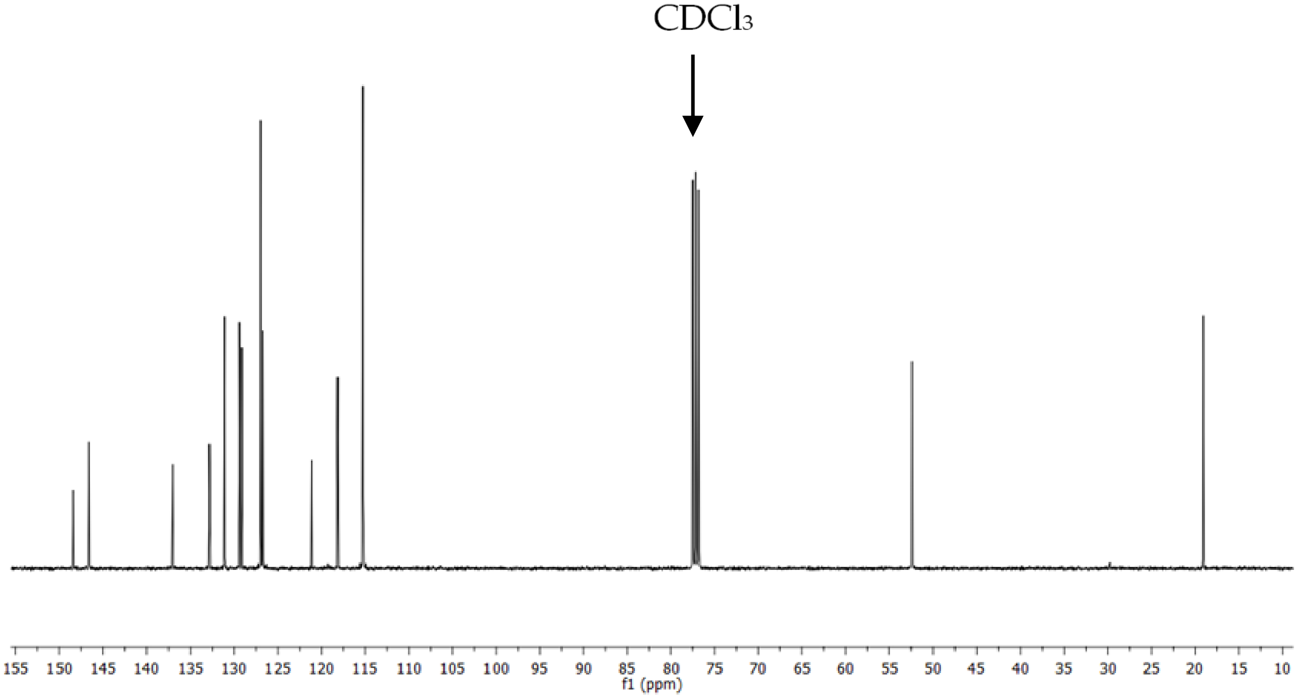
CDCl3
155 150 145 140 135 130 125 120 115 110 105 100 95 90 85 80 75 70 65 60 55 50 45 40 35 30 25 20 15 10
f1 (ppm)

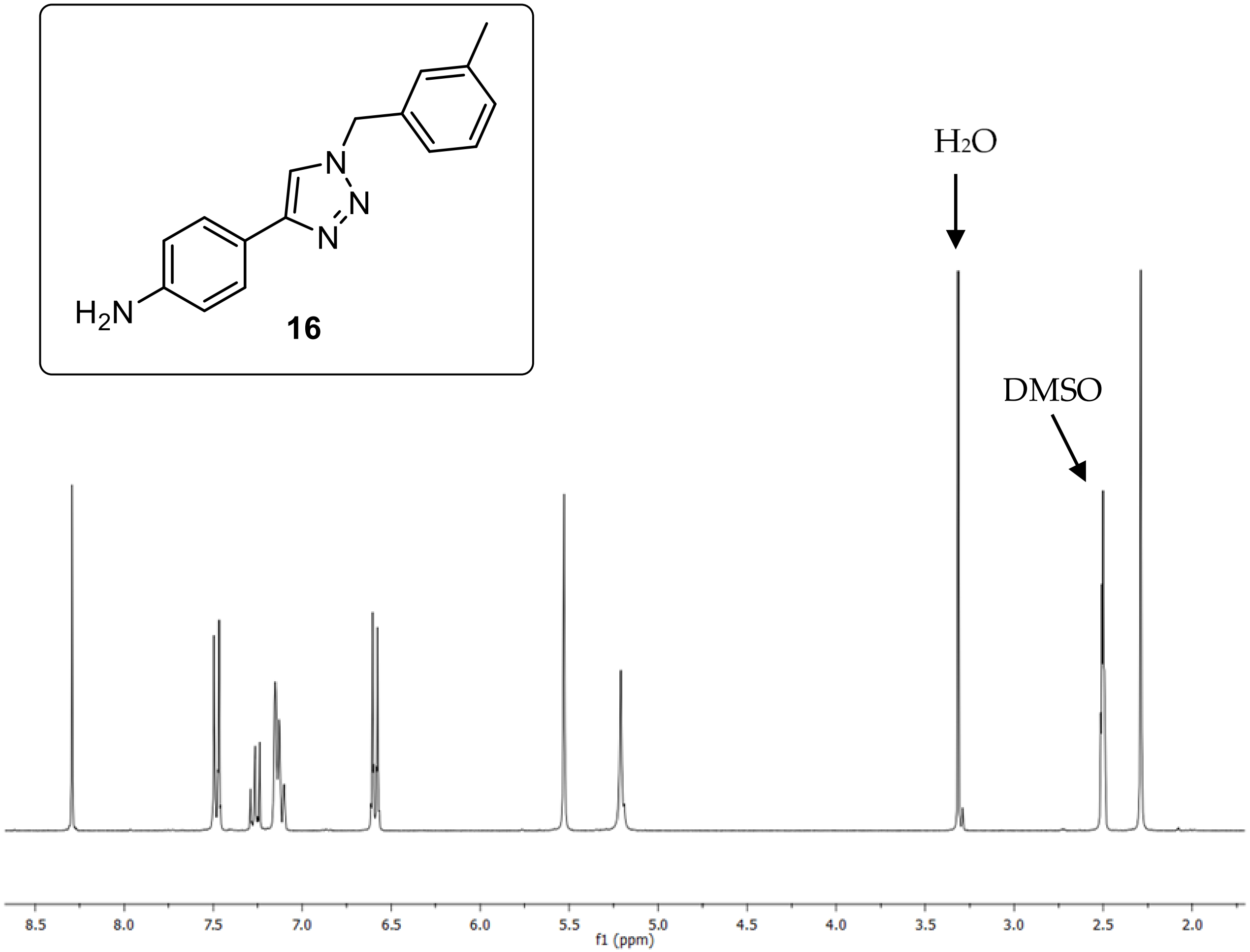
H2N
N
N
N
16
H2O
DMSO
8.5 8.0 7.5 7.0 6.5 6.0 5.5 5.0 4.5 4.0 3.5 3.0 2.5 2.0
f1 (ppm)

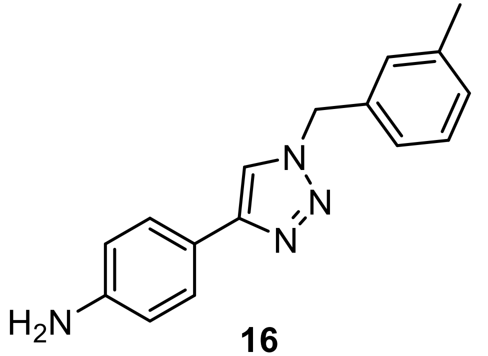
H2N
N
N
N

16

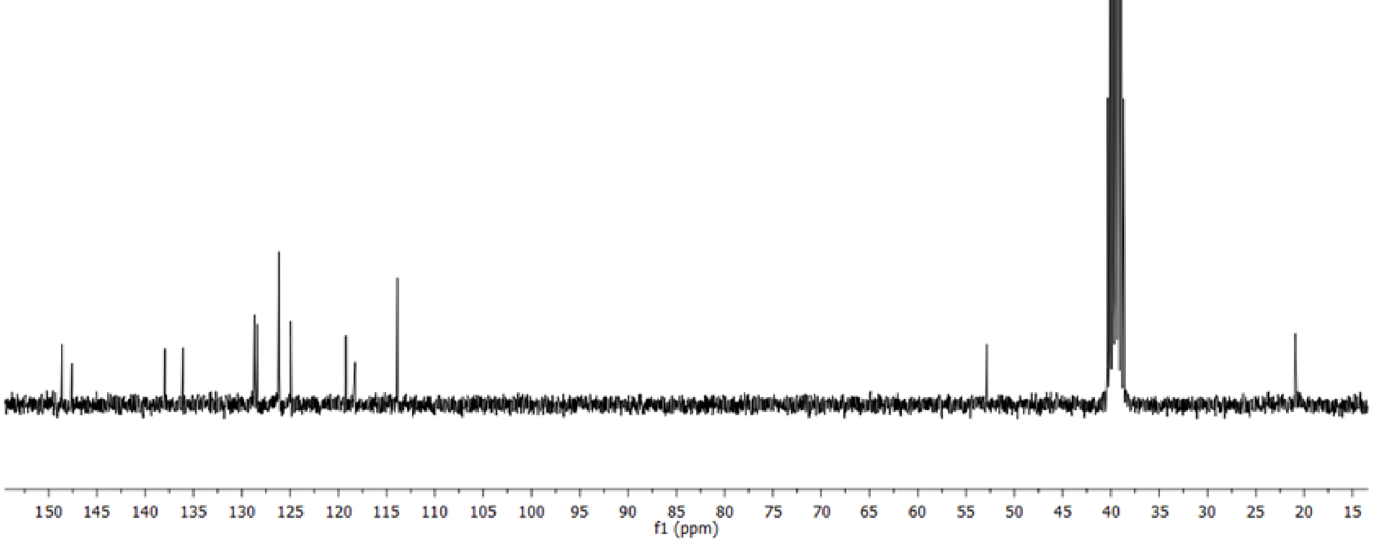
DMSO
150 145 140 135 130 125 120 115 110 105 100 95 90 85 80 75 70 65 60 55 50 45 40 35 30 25 20 15
f1 (ppm)

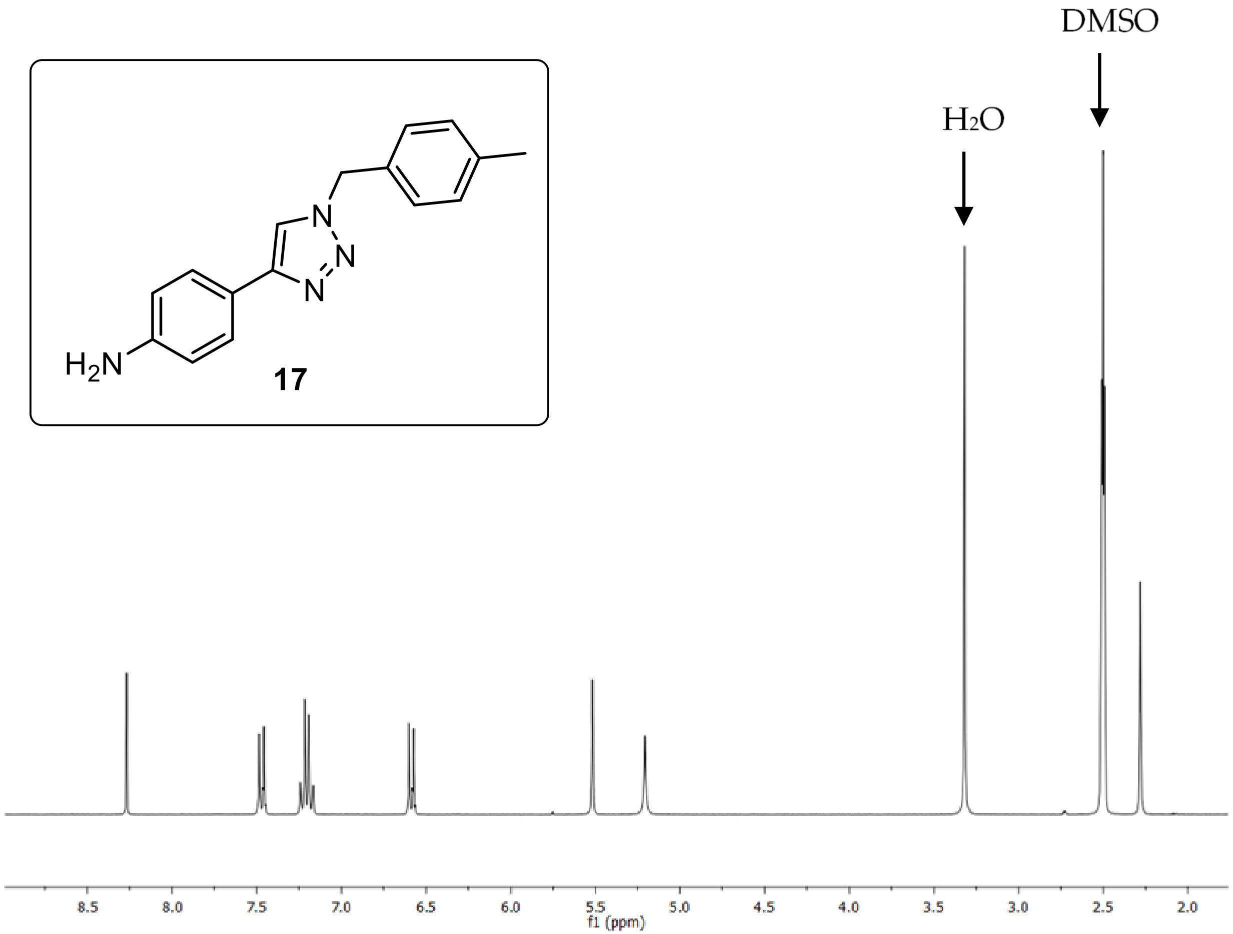

17
H2O
DMSO
8.5
8.0
7.5
7.0
6.5
6.0
5.5
5.0
4.5
4.0
3.5
3.0
2.5
2.0
f1 (ppm)

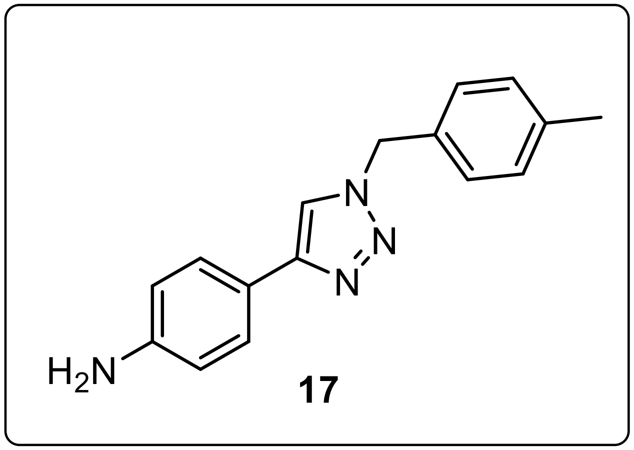
H2N
N
N
N
17

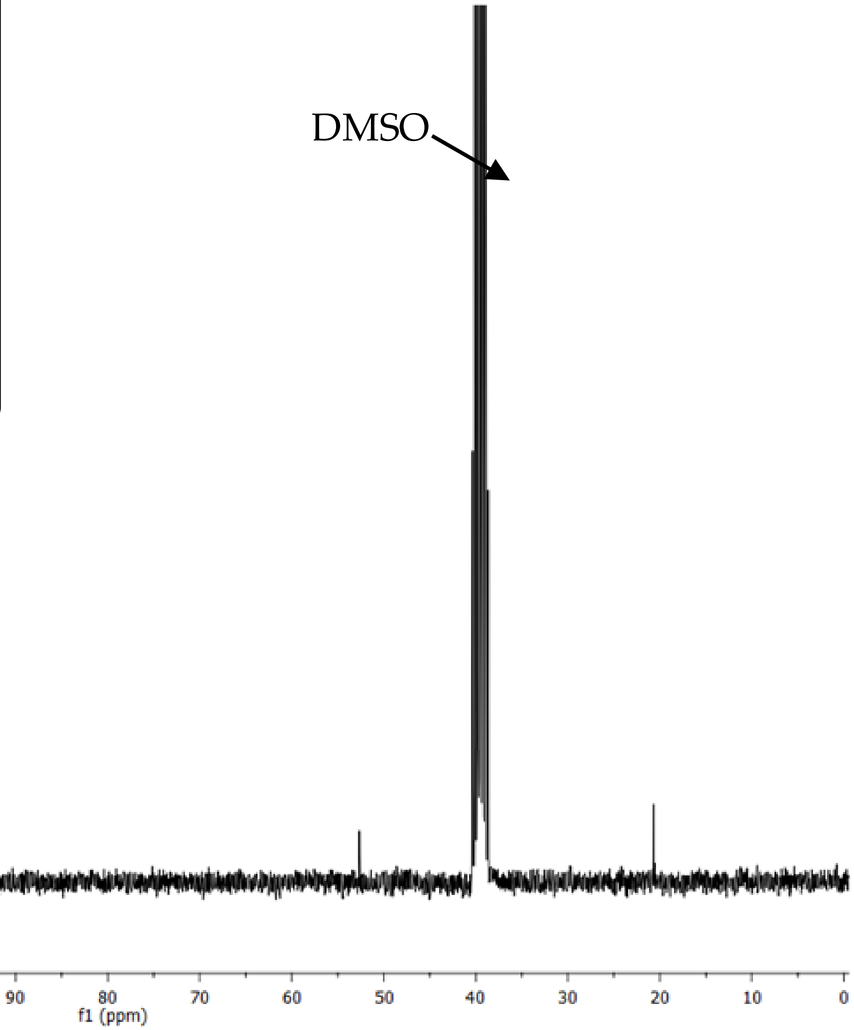
DMSO
150
140
130
120
110
100
90
80
70
60
50
40
30
20
10
0
f1 (ppm)

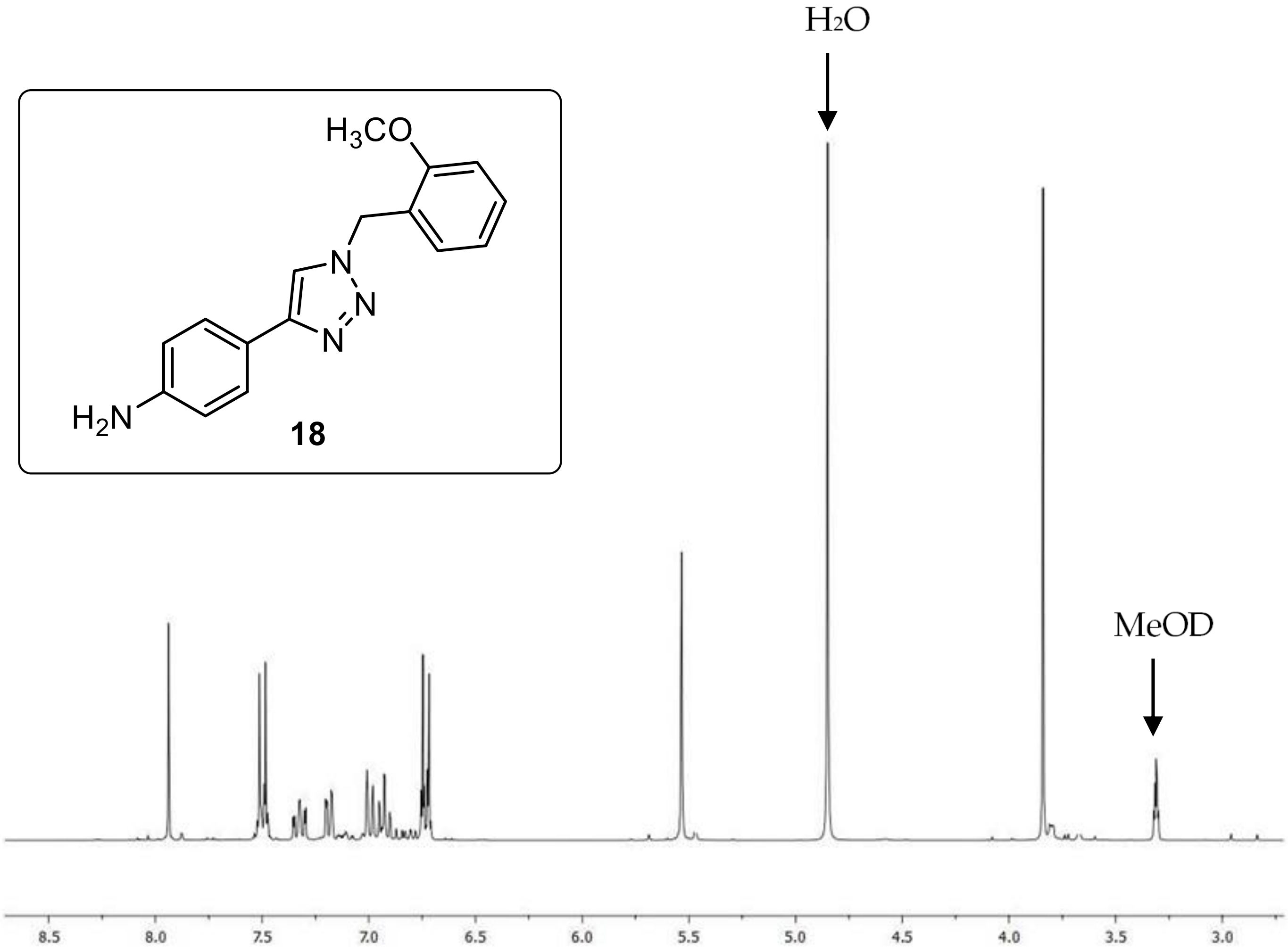
H2O
H3CO
N
N
N
H2N
18
MeOD
8.5
8.0
7.5
7.0
6.5
6.0
5.5
5.0
4.5
4.0
3.5
3.0

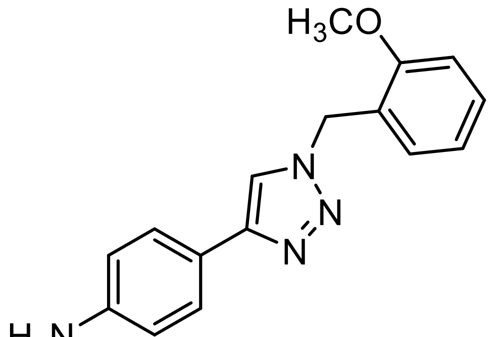
H3CO
N
N
N
H2N

18

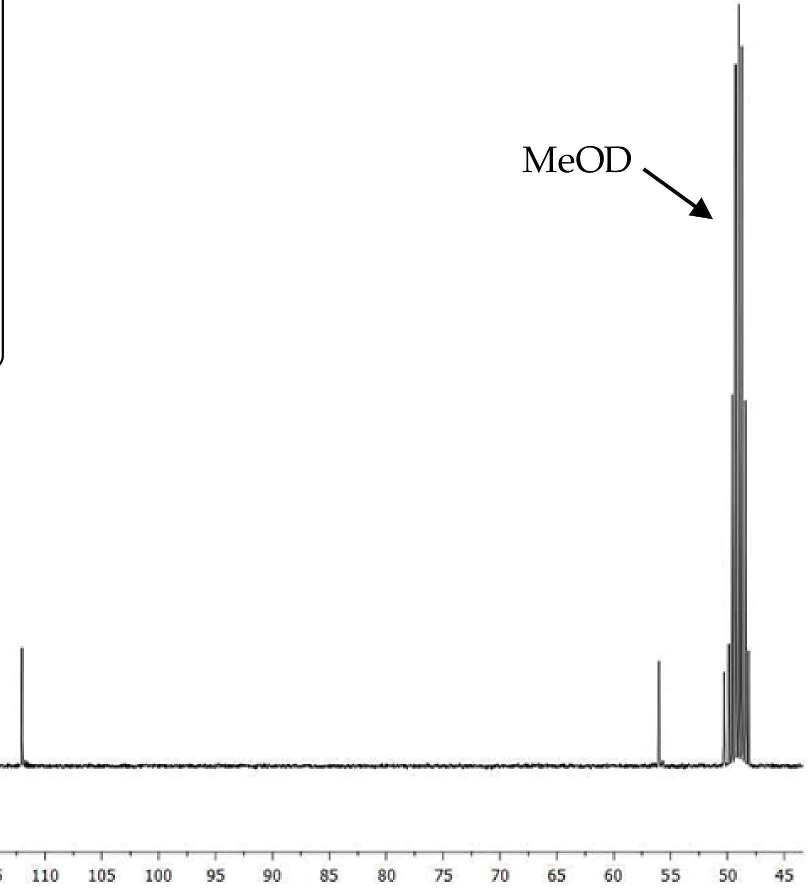
MeOD
165 160 155 150 145 140 135 130 125 120 115 110 105 100 95 90 85 80 75 70 65 60 55 50 45

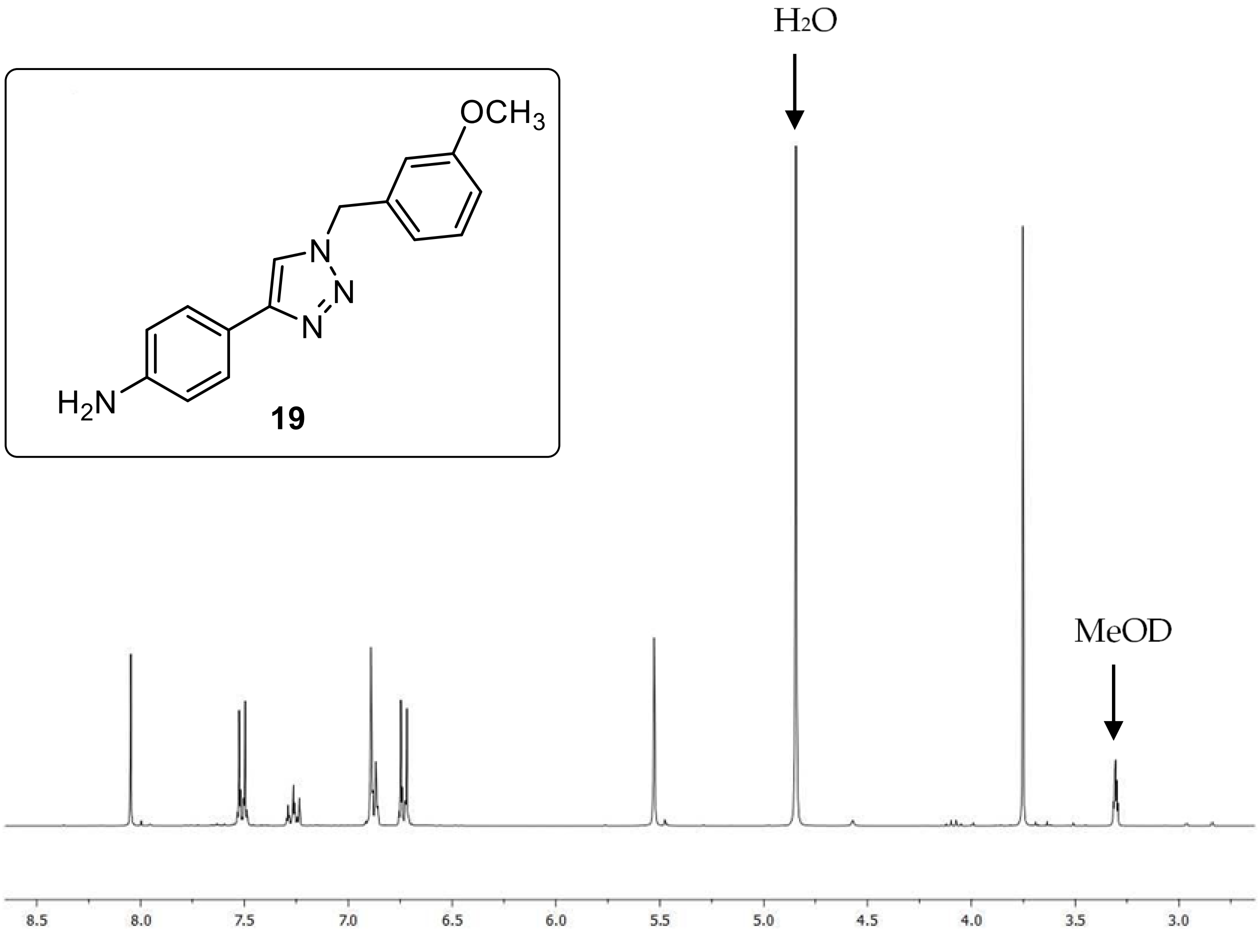
H2O
OCH3
H2N
19
MeOD
8.5
8.0
7.5
7.0
6.5
6.0
5.5
5.0
4.5
4.0
3.5
3.0

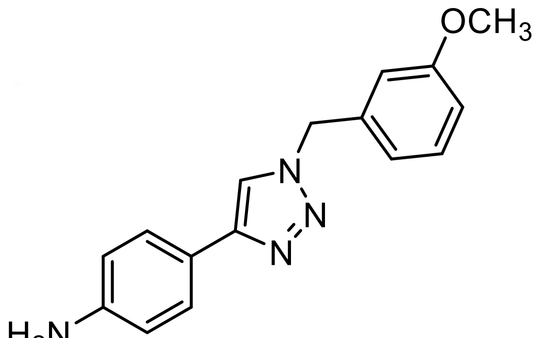

19

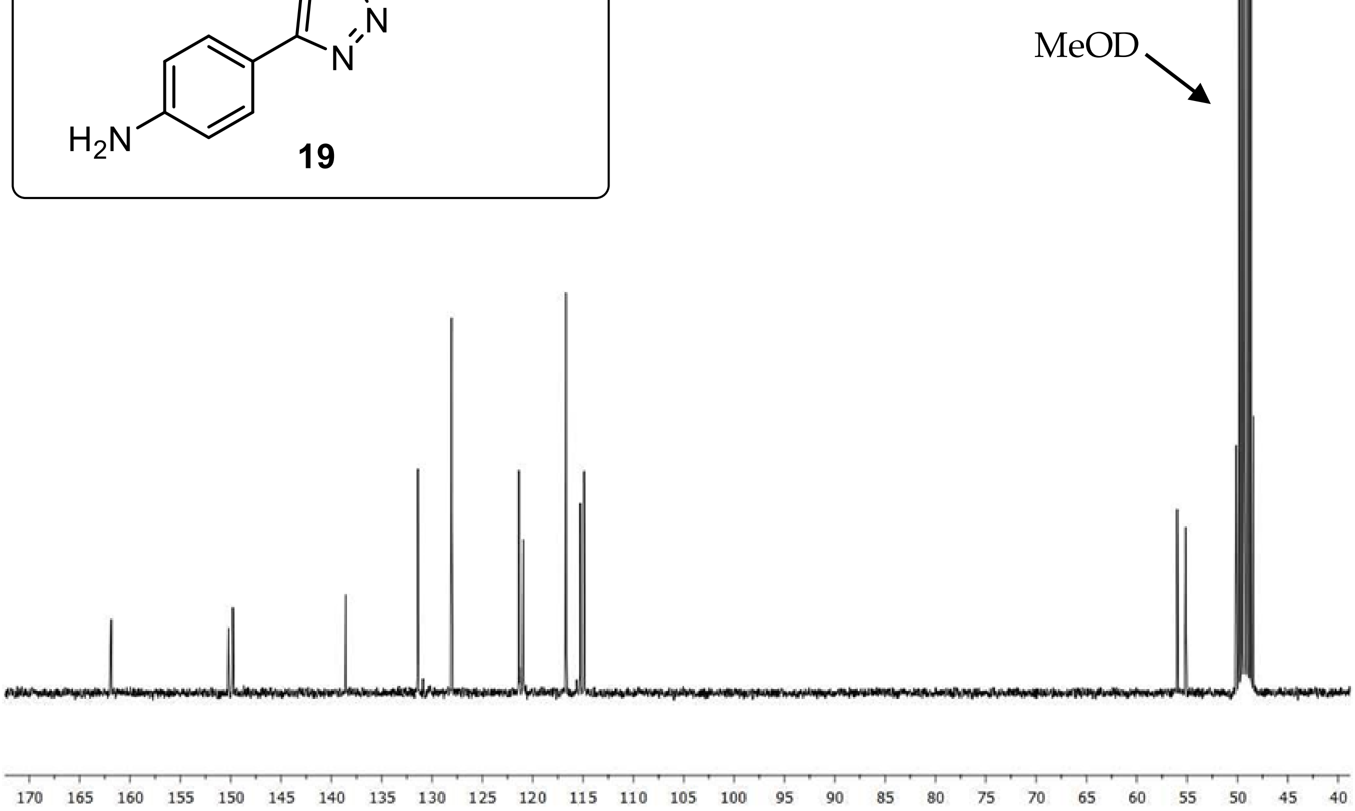
MeOD
170 165 160 155 150 145 140 135 130 125 120 115 110 105 100 95 90 85 80 75 70 65 60 55 50 45 40

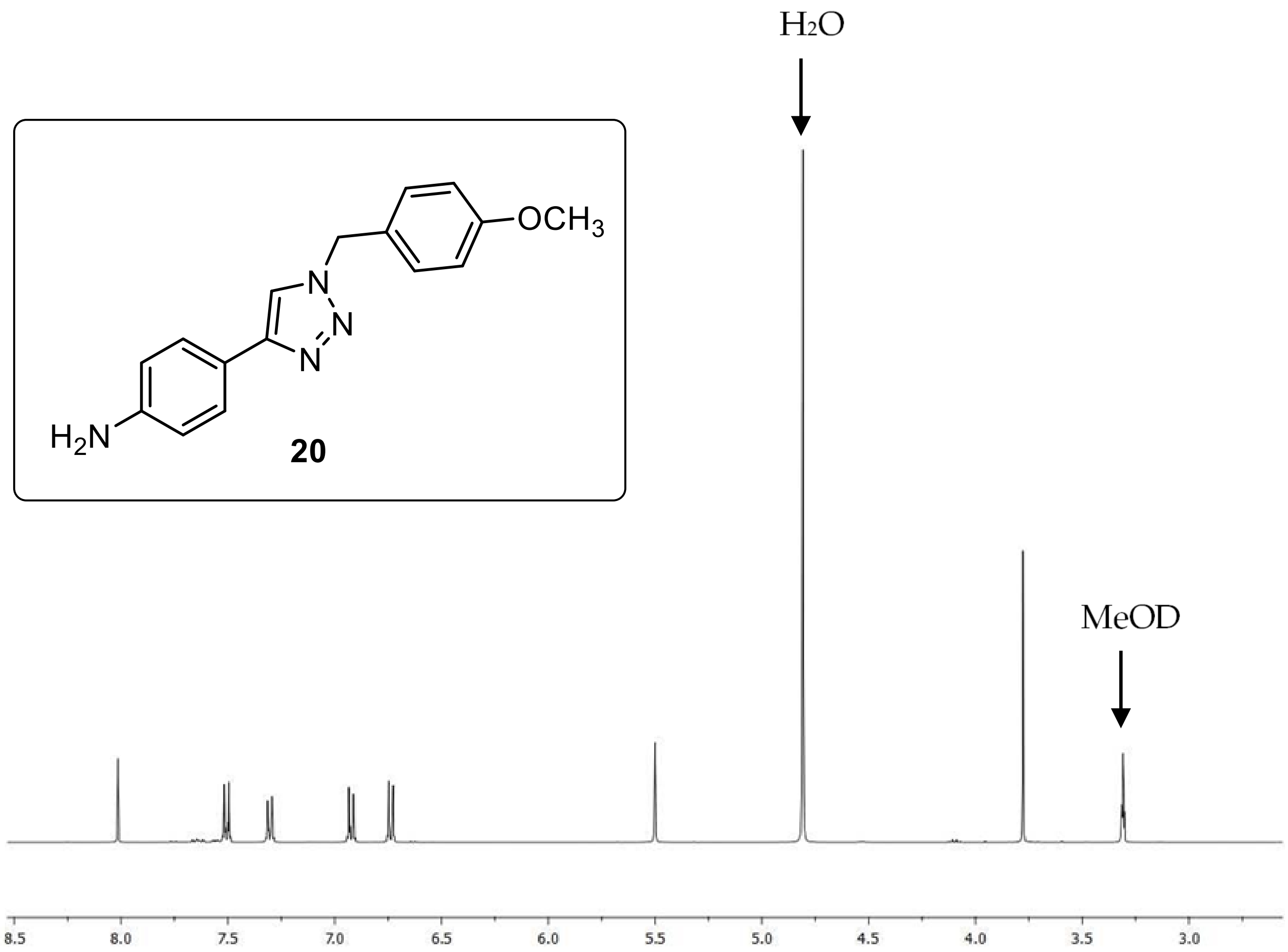
H2O
MeOD
OCH3
H2N
N
N
N
20
8.5
8.0
7.5
7.0
6.5
6.0
5.5
5.0
4.5
4.0
3.5
3.0

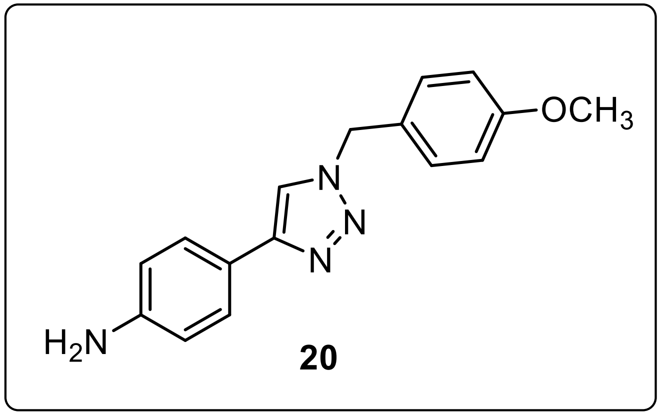
OCH3
N
N
N
H2N
20

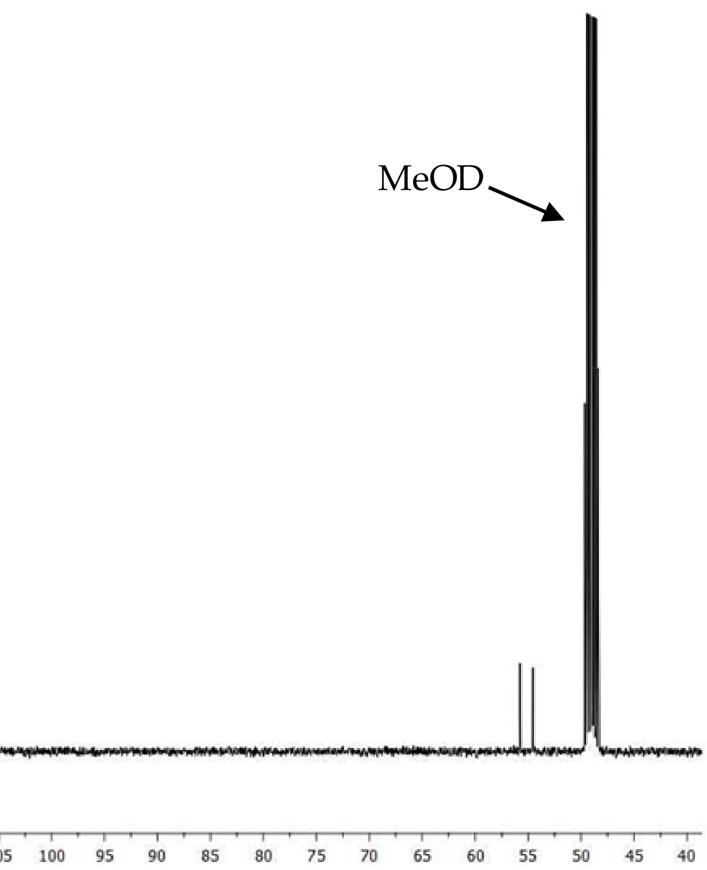
MeOD
165 160 155 150 145 140 135 130 125 120 115 110 105 100 95 90 85 80 75 70 65 60 55 50 45 40

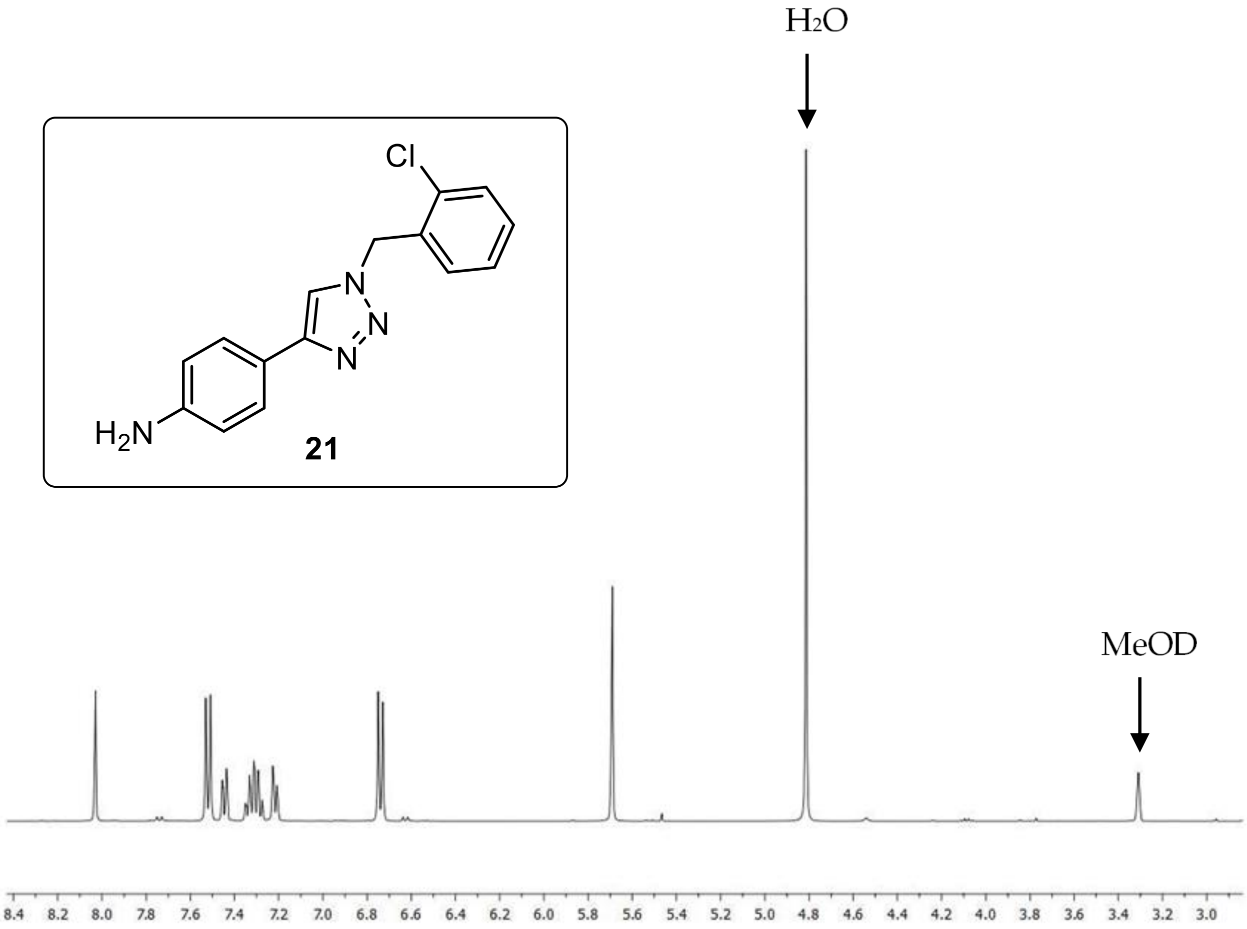
H2O
MeOD
Cl
N
N
N
H2N
21
8.4 8.2 8.0 7.8 7.6 7.4 7.2 7.0 6.8 6.6 6.4 6.2 6.0 5.8 5.6 5.4 5.2 5.0 4.8 4.6 4.4 4.2 4.0 3.8 3.6 3.4 3.2 3.0

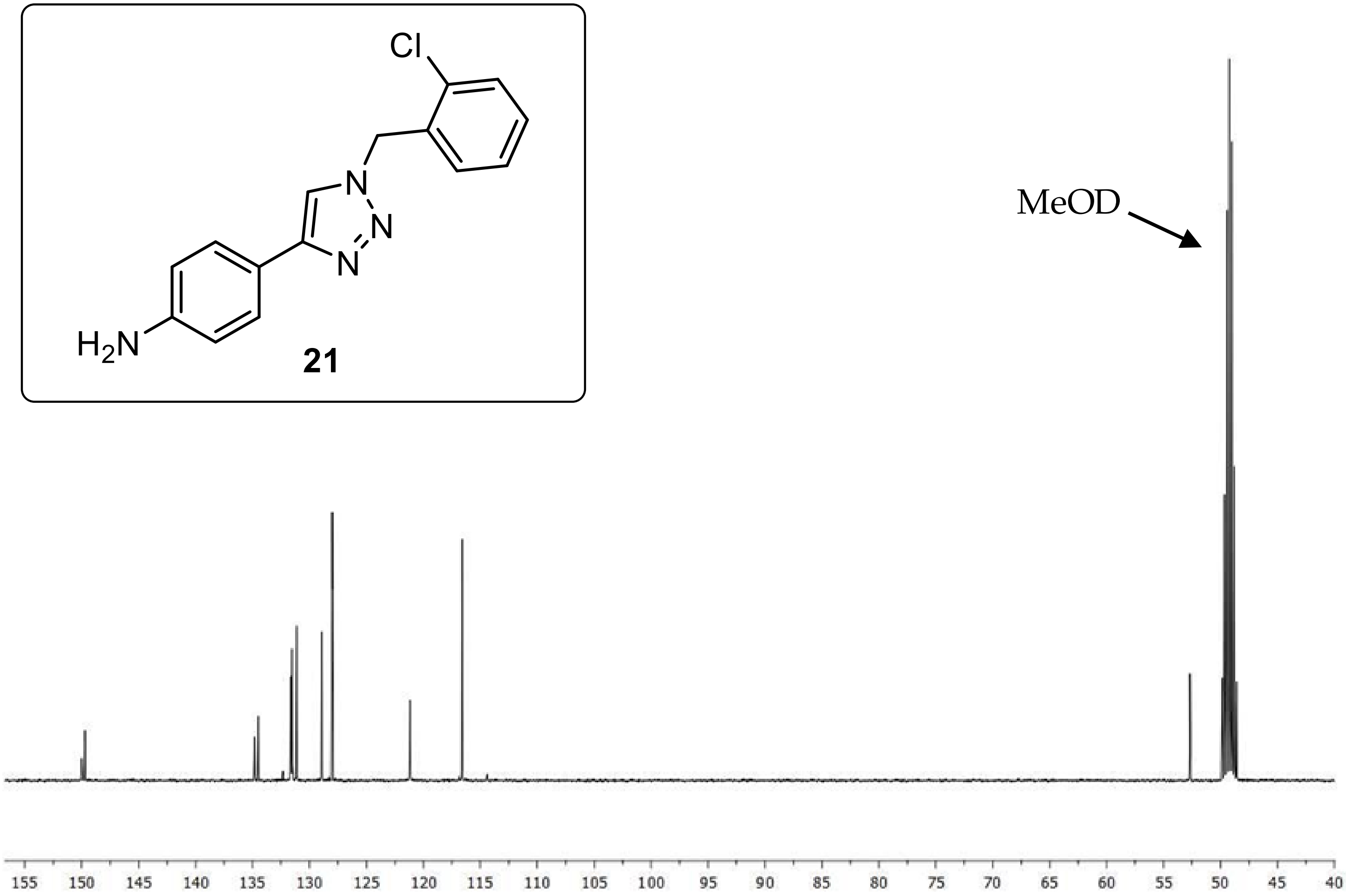
Cl
N
N
N
H_2N
21
MeOD
155 150 145 140 135 130 125 120 115 110 105 100 95 90 85 80 75 70 65 60 55 50 45 40

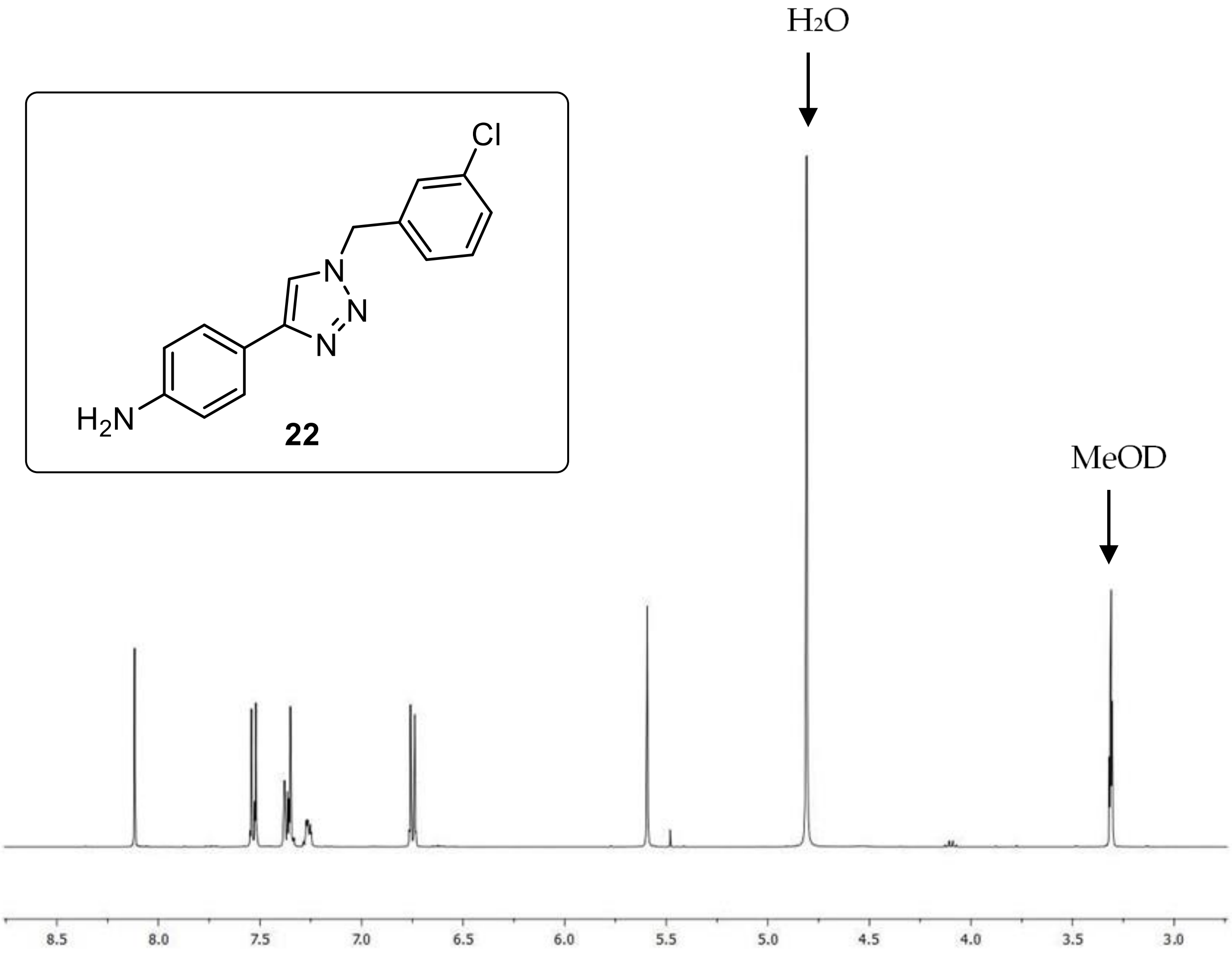
H2O
MeOD
Cl
N
N
N
H2N
22
8.5
8.0
7.5
7.0
6.5
6.0
5.5
5.0
4.5
4.0
3.5
3.0

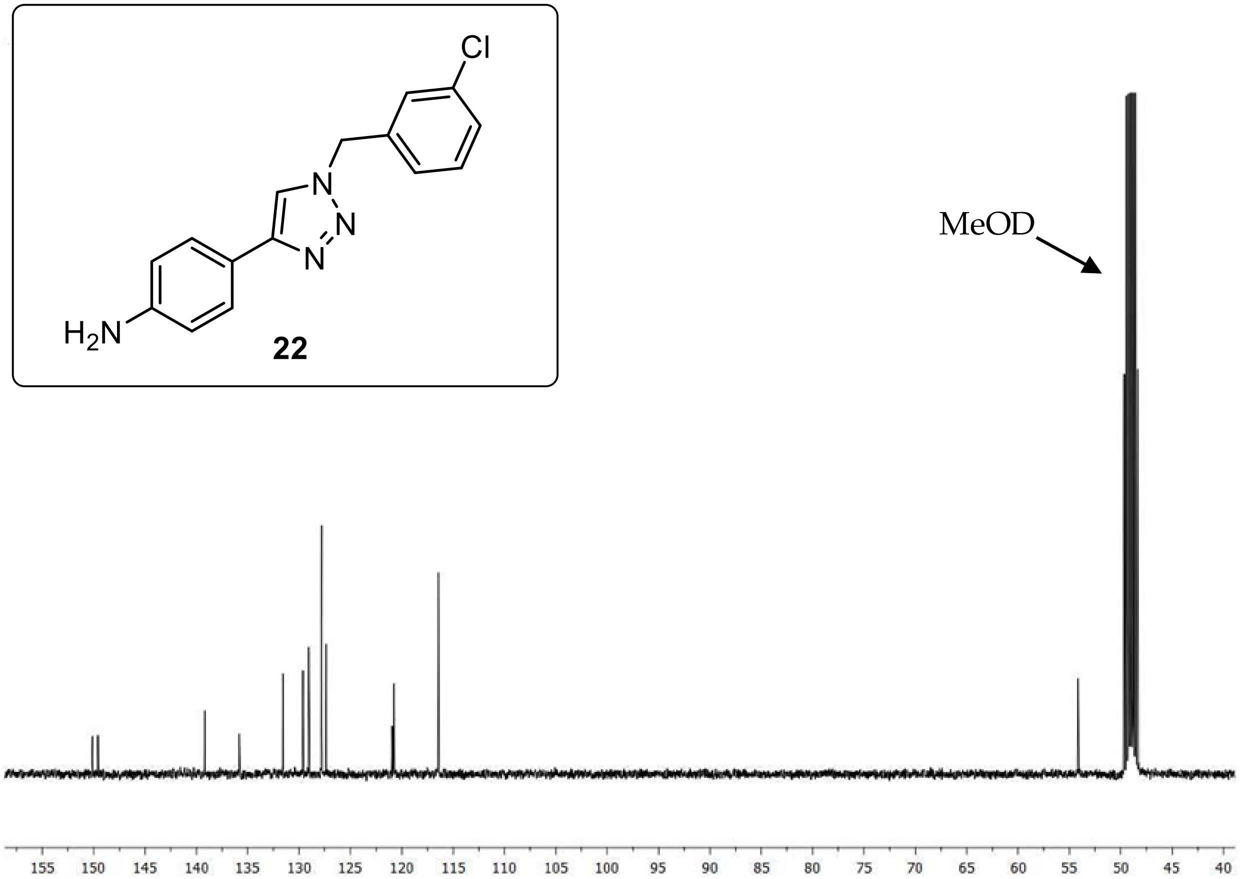
22
MeOD
155 150 145 140 135 130 125 120 115 110 105 100 95 90 85 80 75 70 65 60 55 50 45 40

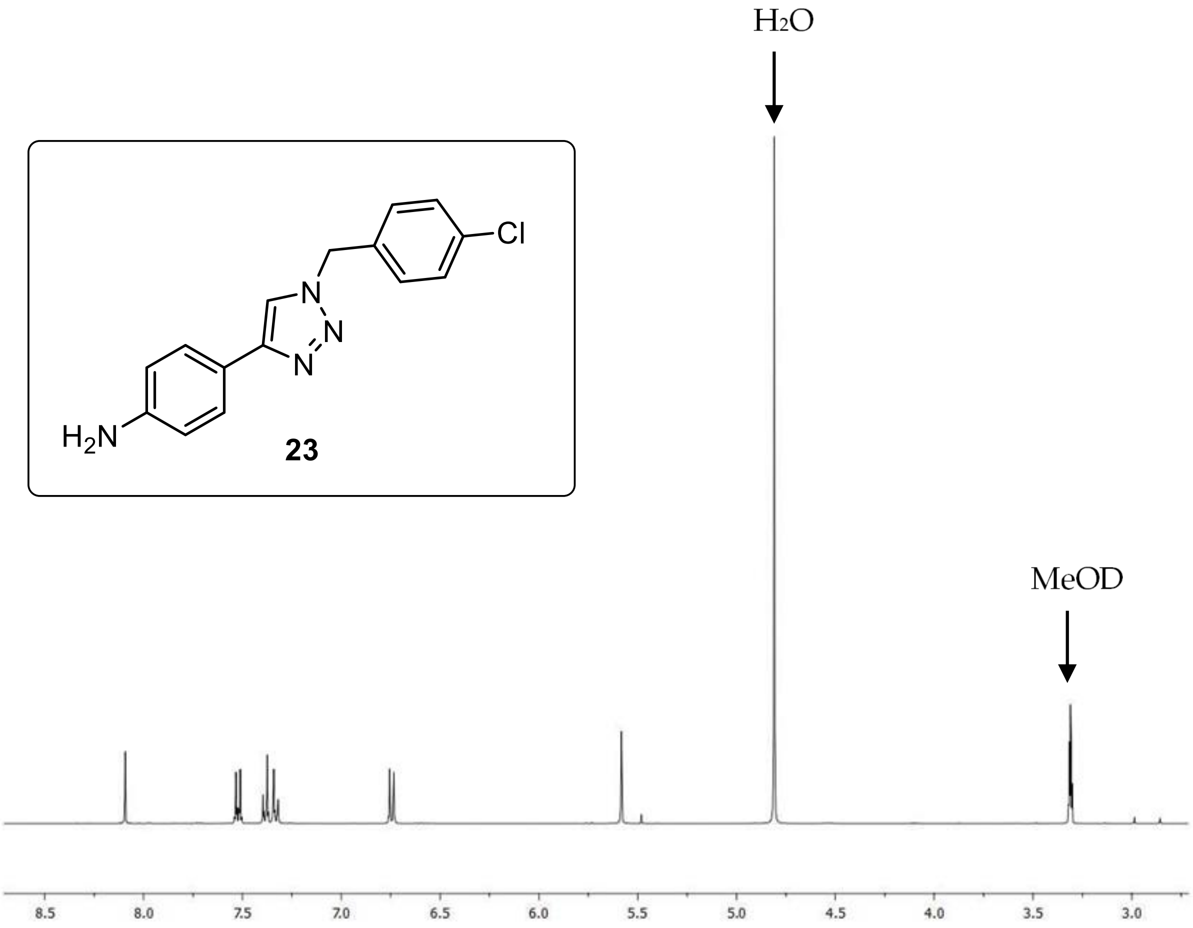
H2O
MeOD
Cl
N
N
N
H2N
23
8.5
8.0
7.5
7.0
6.5
6.0
5.5
5.0
4.5
4.0
3.5
3.0

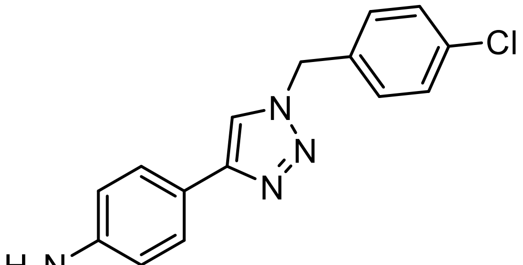
Cl
N
N
N
H2N

23

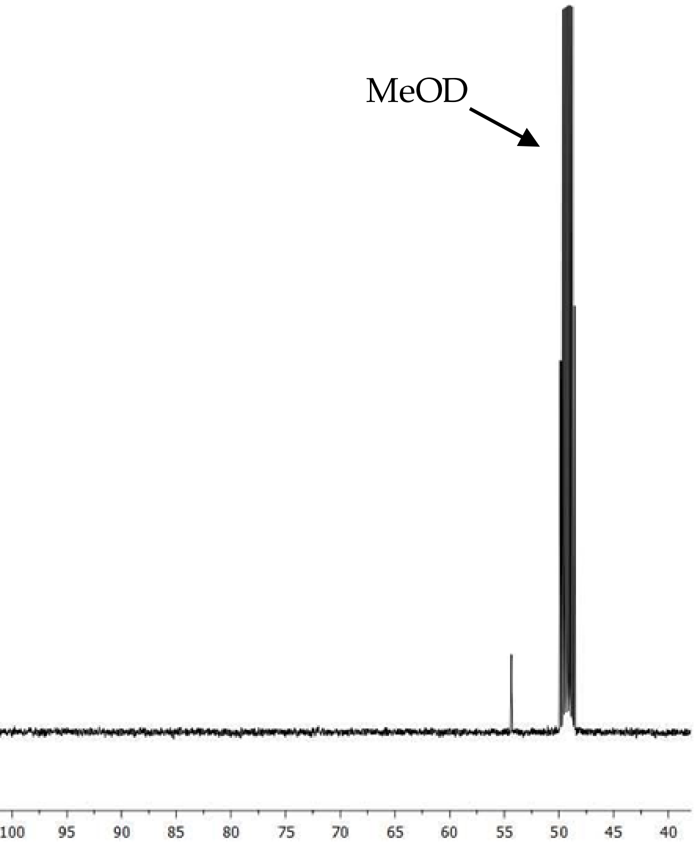
MeOD
155 150 145 140 135 130 125 120 115 110 105 100 95 90 85 80 75 70 65 60 55 50 45 40

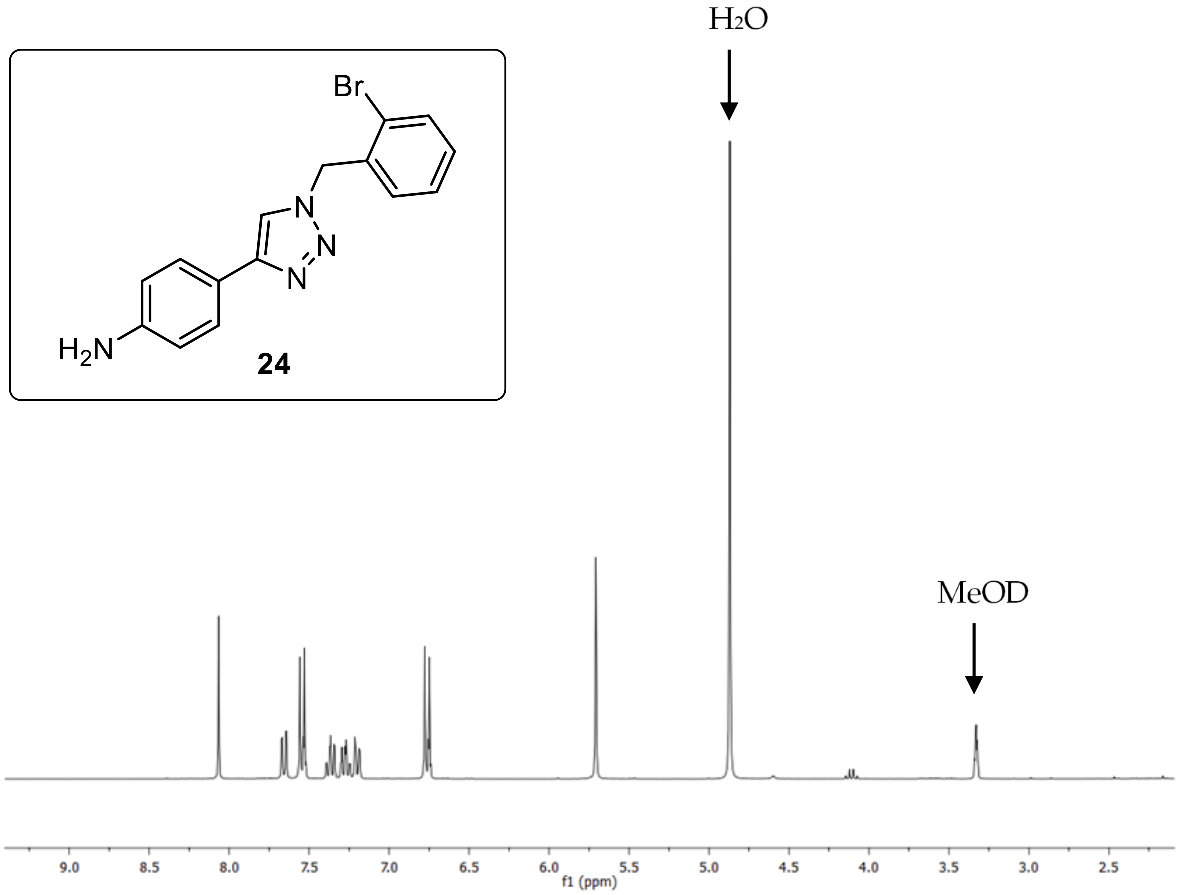
H2O
MeOD
Br
N
N
N
H2N
24
9.0 8.5 8.0 7.5 7.0 6.5 6.0 5.5 5.0 4.5 4.0 3.5 3.0 2.5
f1 (ppm)

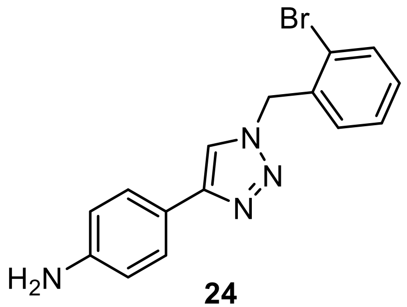
Br
N
N
N
H2N

24

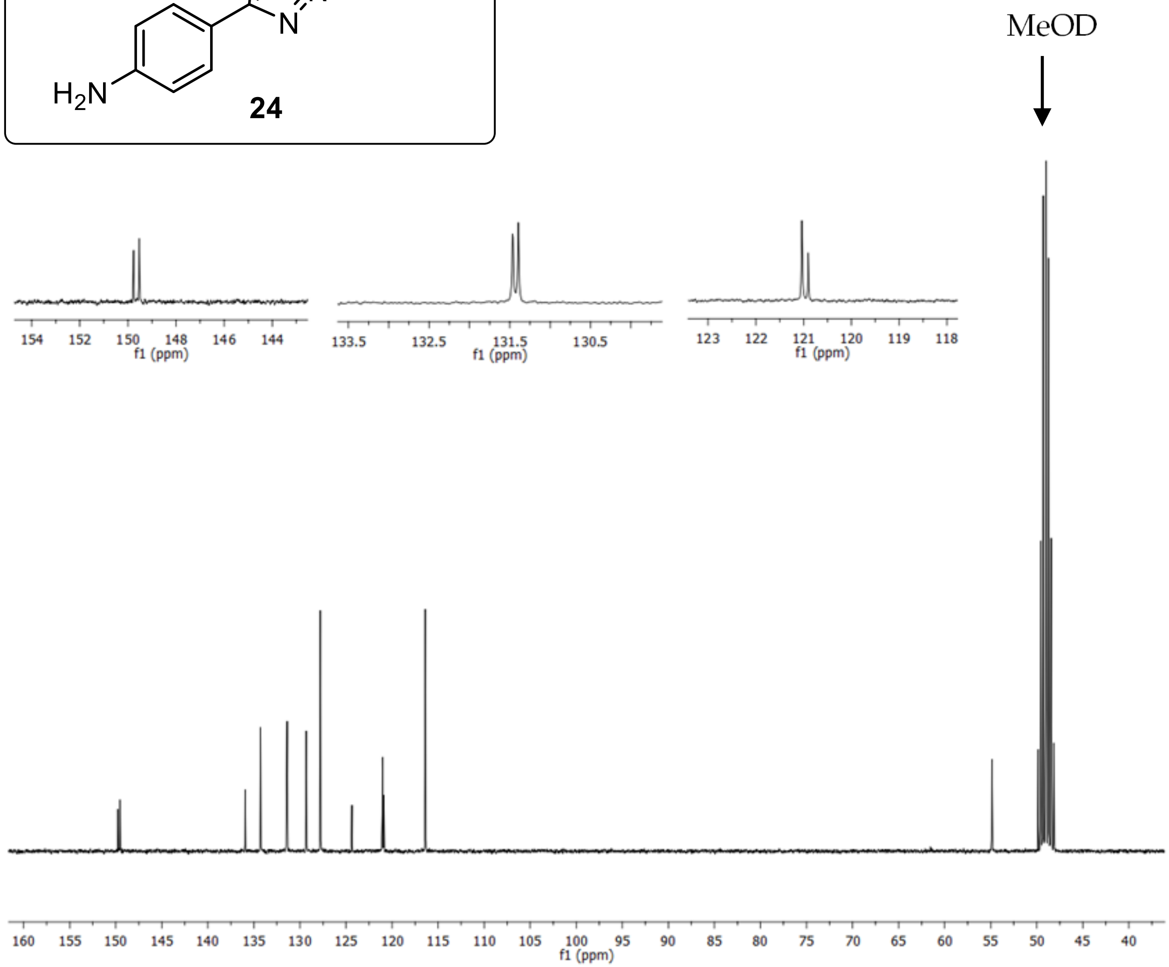
MeOD
154
152
150
148
146
144
f1 (ppm)
133.5
132.5
131.5
130.5
f1 (ppm)
123
122
121
120
119
118
f1 (ppm)
160
155
150
145
140
135
130
125
120
115
110
105
100
95
90
85
80
75
70
65
60
55
50
45
40
f1 (ppm)

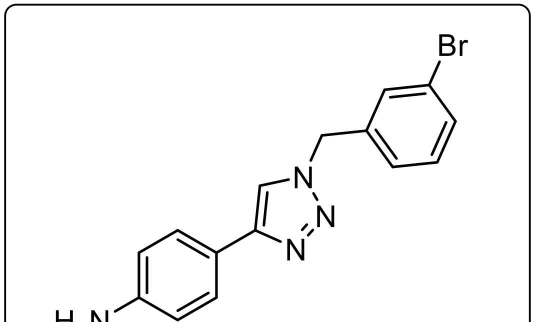

25

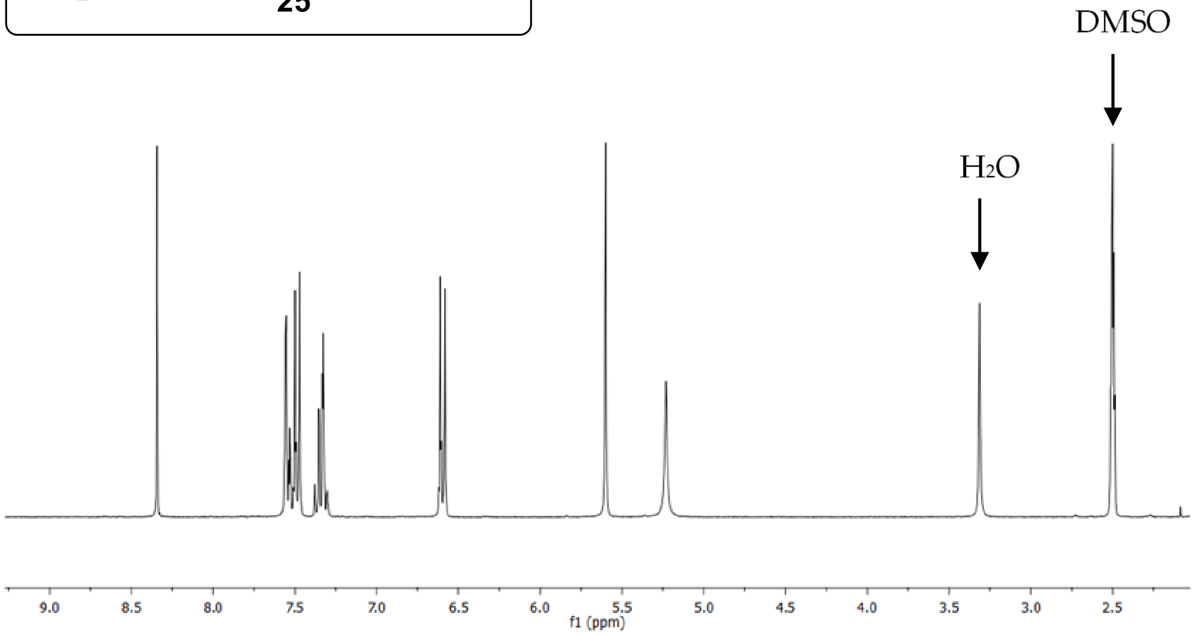

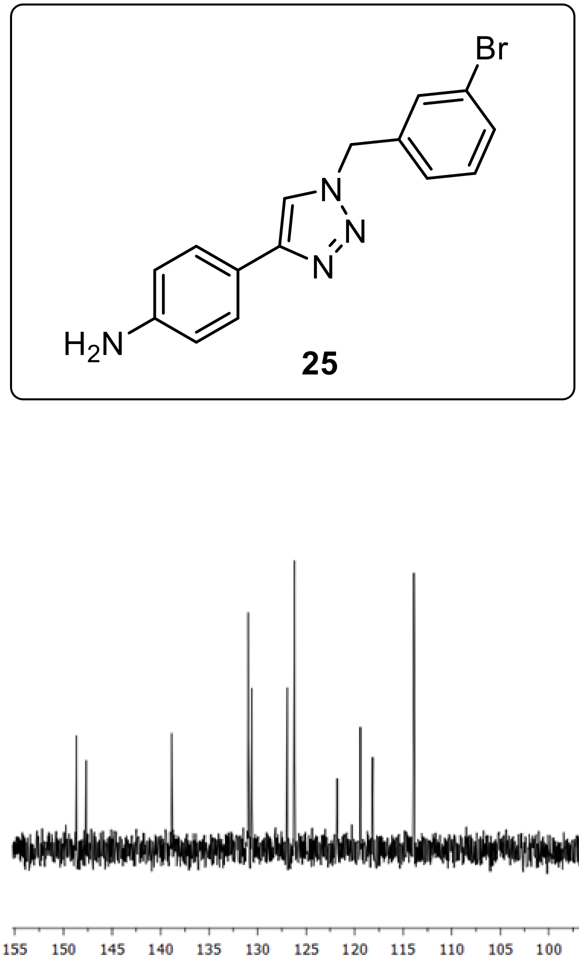
Br
N
N
N
H_2N

25

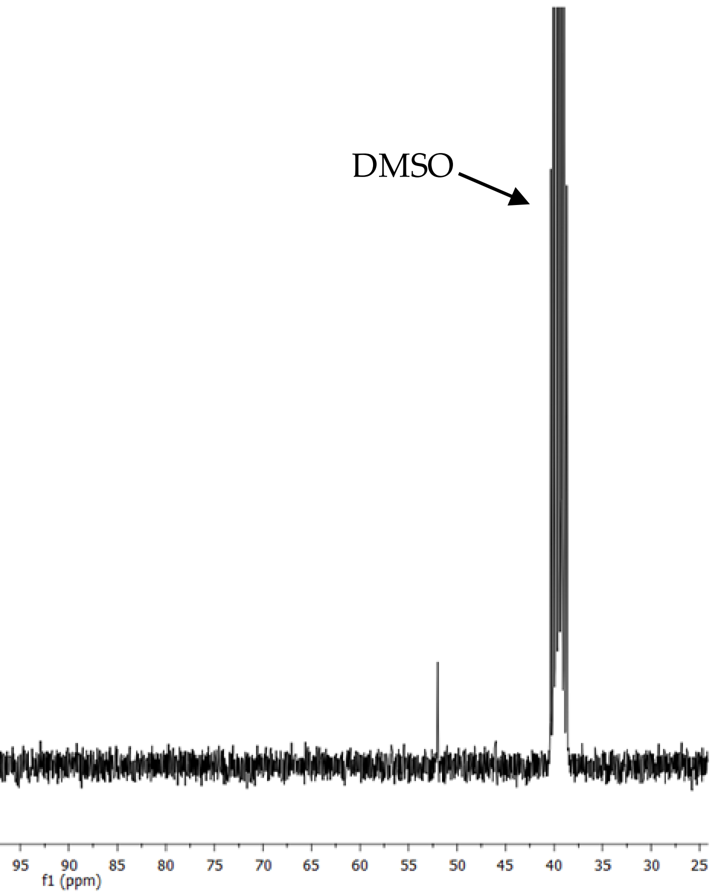
DMSO
155 150 145 140 135 130 125 120 115 110 105 100 95 90 85 80 75 70 65 60 55 50 45 40 35 30 25
f1 (ppm)

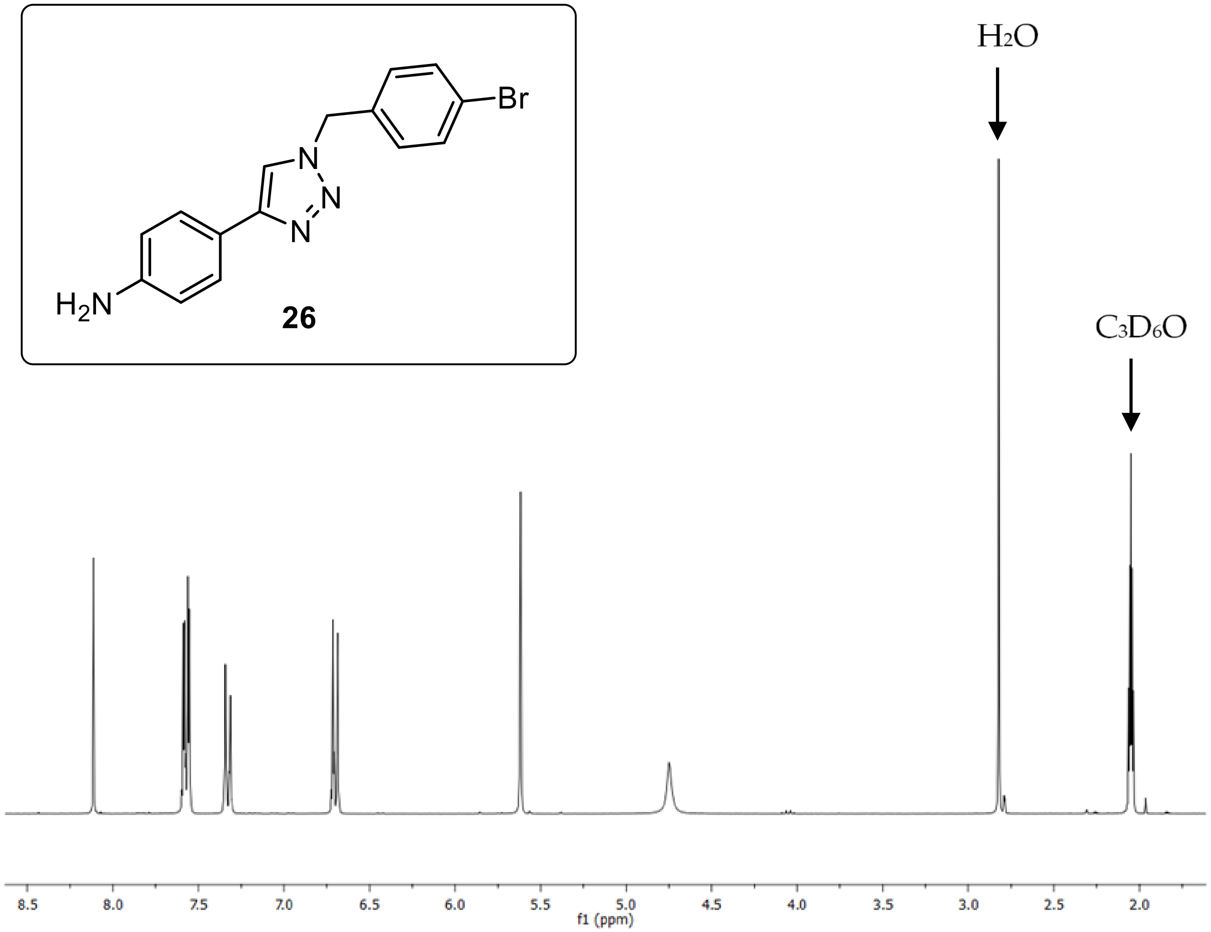
Br
H2N
26
H2O
C3D6O
8.5
8.0
7.5
7.0
6.5
6.0
5.5
5.0
4.5
4.0
3.5
3.0
2.5
2.0
f1 (ppm)

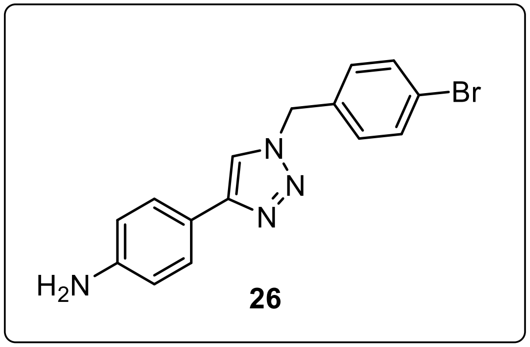

Br
N
N
N
H$_2$N
26

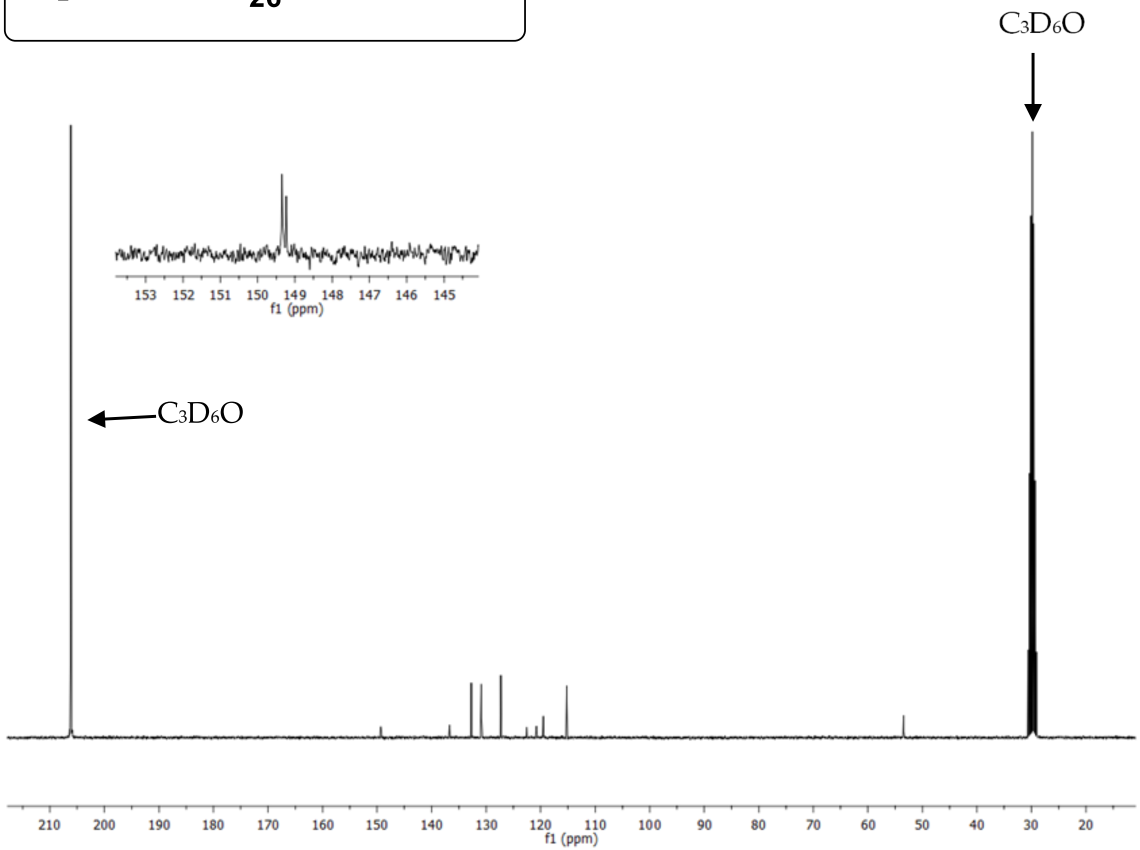

C$_3$D$_6$O
C$_3$D$_6$O
153 152 151 150 149 148 147 146 145
f1 (ppm)
210 200 190 180 170 160 150 140 130 120 110 100 90 80 70 60 50 40 30 20
f1 (ppm)
